# Bioinformatics analysis and experimental verification of the prognostic and biological significance mediated by fatty acid metabolism related genes for hepatocellular carcinoma

**DOI:** 10.3389/fonc.2022.972744

**Published:** 2022-08-02

**Authors:** Xiao-Ren Zhu, Jia-Qi Zhu, Yu-Fei Chen, Yuan-Yuan Liu, Jing-Jing Lu, Jun Sun, Shi-Qing Peng, Min-Bin Chen, Yi-Ping Du

**Affiliations:** ^1^ Department of Radiotherapy and Oncology, Affiliated Kunshan Hospital of Jiangsu University, Kunshan, China; ^2^ Department of Medical Oncology, Affiliated Kunshan Hospital of Jiangsu University, Medical School of Jiangsu University, Kunshan, China; ^3^ Department of Thoracic Surgery, Affiliated Hospital of Nantong University, Medical School of Nantong University, Nantong, China; ^4^ Nantong Key Laboratory of Translational Medicine in Cardiothoracic Diseases and Research Institution of Translational Medicine in Cardiothoracic Diseases, Affiliated Hospital of Nantong University, Medical School of Nantong University, Nantong, China; ^5^ Department of Hepatology, Infectious Diseases Hospital Affiliated with Soochow University, Suzhou, China; ^6^ Clinical Research and Lab Center, Affiliated Kunshan Hospital of Jiangsu University, Kunshan, China

**Keywords:** fatty acid metabolism, hepatocellular carcinoma, prognosis model, tumor microenvironment, immunotherapy, HAVCR1

## Abstract

**Background:**

Liver cancer is among the leading causes of death related to cancer around the world. The most frequent type of human liver cancer is hepatocellular carcinoma (HCC). Fatty acid (FA) metabolism is an emerging hallmark that plays a promoting role in numerous malignancies. This study aimed to discover a FA metabolism-related risk signature and formulate a better model for HCC patients’ prognosis prediction.

**Methods:**

We collected mRNA expression data and clinical parameters of patients with HCC using the TCGA databases, and the differential FA metabolism-related genes were explored. To create a risk prognostic model, we carried out the consensus clustering as well as univariate and multivariate Cox regression analyses. 16 genes were used to establish a prognostic model, which was then validated in the ICGC dataset. The accuracy of the model was performed using receiver operating characteristic (ROC) analyses, decision curve analysis (DCA) and nomogram. The immune cell infiltration level of risk genes was evaluated with single-sample GSEA (ssGSEA) algorithm. To reflect the response to immunotherapy, immunophenoscore (IPS) was obtained from TCGA-LIHC. Then, the expression of the candidate risk genes (p < 0.05) was validated by qRT-PCR, Western blotting and single-cell transcriptomics. Cellular function assays were performed to revealed the biological function of HAVCR1.

**Results:**

According to the TCGA-LIHC cohort analysis, the majority of the FA metabolism-related genes were expressed differentially in the HCC and normal tissues. The prognosis of patients with high-risk scores was observed to be worse. Multivariate COX regression analysis confirmed that the model can be employed as an independent prognosis factor for HCC patients. Furthermore, ssGSEA analysis revealed a link between the model and the levels of immune cell infiltration. Our model scoring mechanism also provides a high predictive value in HCC patients receiving anti-PDL1 immunotherapy. One of the FA metabolism-related genes, HAVCR1, displays a significant differential expression between normal and HCC cell lines. Hepatocellular carcinoma cells (Huh7, and HepG2) proliferation, motility, and invasion were all remarkably inhibited by HAVCR1 siRNA.

**Conclusion:**

Our study identified a novel FA metabolism-related prognostic model, revealing a better potential treatment and prevention strategy for HCC.

## Introduction

Hepatocellular carcinoma (HCC) is the most widely known malignancy, resulting in significant human mortalities (1). The 5-year overall survival (OS) of patients with HCC has decreased by 20% globally and by 12% in Asian countries (2). Patients with advanced metastatic and/or recurrent HCCs have failed to gain benefit over the long term from standard HCC treatments such as surgical resection and liver transplantation (3). Therefore, it is crucial to explore novel molecularly-targeted therapies and new prognostic factors for HCC patients (4–6). Very, recently, multiple personalized molecular subtypes of HCC have been reported. Fu et al. constructed a novel predictive model for the prognosis of patients with HCC based on pyroptosis-related genes by categorizing HCC patients into two subgroups from the TCGA dataset (7). However, the accuracy of prognosis for HCC patients is still poor. Hence, more efficient prognostic factors must be explored.

Metabolic abnormalities are a typical characteristic of cancer (8). Cancer cells have unique metabolic characteristics that distinguish them from normal cells. When carcinogenic signals are blocked, cancer cells may be able to survive in the adverse microenvironments by metabolic reprogramming (9). Increasing evidence supports the critical involvement of metabolic reprogramming in tumor onset and progression (10–12). FA metabolism disorder has become a typical cancer cell characteristic (13). Many cellular biological processes require FAs, including membrane formation, signaling molecule release, and energy storage. FAs are essential for cancer formation and progression, according to several studies (14, 15). Wang et al. demonstrated that abnormal activation of various oncogenic signaling cascades promotes HCC development by regulating the lipid-metabolizing enzyme expression and/or activity, as well as FA metabolism reprogramming (16). Although FA metabolism has been associated with HCC oncogenesis, its correlation with the progression and clinical prognosis of HCC is yet unknown. Thus, the identification of novel and valuable characteristic molecular models linked to FA metabolism may shed light on the anti-HCC strategy.

The objective of this research was to develop a novel prognostic model according to DEGs linked to FA metabolism and to explore its relationship with clinicopathological parameters and OS in HCC. In addition, the correlation between the tumor immune microenvironment (TIME) and model genes was explored. The potential of our model in guidance of anti-PD-1 immunotherapy was also investigated. Furthermore, the hub gene HAVCR1 was selected for further functional validation of HCC cells *in vitro* based on its expression level. Collectively, our study provides new insight into the relationship between FA metabolism and HCC.

## Materials and methods

### Data acquisition

The RNA-seq data (FPKM format), including 374 HCC and 50 normal tissues, along with clinical data, were provided by the TCGA database. The LIRI-JP cohort data set, containing transcriptomics data from 231 HCC patients, was retrieved from the ICGC database. cBioPortal shows genetic alterations in 16-risk-gene (17). The HPA database is composed of numerous sections that integrate several omics technologies for researchers (18–20). IHC images showed the difference in HAVCR1 protein expression between HCC tissues and normal tissues.

### Analysis of differential expression of genes related to FA metabolism

**Table 1**lists 30 FA metabolism-related genes that were retrieved for the study. Differentially expressed genes (DEGs) in HCC were identified by using the “Limma” package of R (p <0.05) according to the screening criteria.

**Table 1:** 30 FA metabolism-related genes.

SREBF1 ELOVL5 SPTLC3 ACSL1 FABP5 FASN AMACR SPOP DGAT1 ABHD5 PDHA1 ELOVL3 ACOX1 OXSM ACAA2 ADH5 ACACB CPT2 SLC17A2 CPT1C ACADL HADH ELOVL6 ADH6 SIRT1 ACADM ACSL6 FABP4 SCD CD36

We utilized the STRING database to create a protein-protein interaction network (PPI) regarding DEGs, and Cytoscape helped us visualize the interactive network data. The mutual regulatory relationship between DEGs was demonstrated by R (version 4.1.2).

### Consensus clustering and functional enrichment analysis

We employed the R package “Consensus ClusterPlus”, for consensus clustering, and the mRNA expression data of 30 genes, which were highly correlated with FA metabolism, were classified into several molecular subtypes *via* the K-means clustering (21). 1,000 iterations were carried out to make sure the classification was accurate. DEGs were screened for further analysis based on the samples from the prior cluster analysis (|log2FC| >1, adjusted p <0.05). R package “GOplot” and “ggplot2” were used to perform GO and KEGG analyses between the two groups (22).

### Construction and validation of FA metabolism-related prognostic model

Initially, we used the univariate cox analysis for DEGs to identify and screen out the genes that were associated with prognosis in HCC patients (p < 1*10-6). These genes were then subjected to multivariate Cox analysis to identify 16 genes linked to prognosis, and the prognostic risk assessment construction formula is as stated below:

Risk score = coefficient1 * expression of gene1 +… + coefficientN * expression of geneN

As per the median risk scores, patients were subsequently classified into two groups. The OS of HCC patients in both groups was assessed through KM analysis using the “Survival” and “SurvMiner” R packages. The “Rtsne” package was used to perform PCA analysis on 16 prognostic genes to reduce the dimension of complex data. For identification and comparison of potential prognostic factors, the univariate Cox analysis was used, while a multivariate Cox analysis was performed to test whether the risk score was an independent prognostic factor. The accuracy evaluation of the prognostic model was done by ROC curves using the R packages “SurvivalROC” and “timeROC”. Using the “rms” and “survival” packages, a predictive nomogram was developed according to the risk score and clinicopathological parameters (23). Using the “ggDCA” package, the DCA model intuitively described the relationship between risk score and other parameters.

### GSEA and GSVA

GSEA was employed in our study to investigate potential signaling pathways between the two groups to show a possible molecular mechanism underlying the prognostic difference. The Type and Replacement were set to “phenotype” and “1000”, respectively. “c2.cp.kegg.v6.2.-symbols” was downloaded to perform GSVA, which converts gene alterations into signaling pathway changes. To investigate potential changes in biological function and then annotate different risk genes, the GSVA algorithm and the “clusterProfiler” R package were employed.

### Evaluation of tumor immune microenvironment

Initially, expression data (ESTIMATE) was utilized to quantify the ratio of stromal cells to immune cells in malignancies, which was then used to estimate the TIME in HCC samples indirectly (24). The normalized enrichment score (NES) was then used to calculate the levels of immune function pathway enrichment. The scores of TIME cells were evaluated by the ssGSEA algorithm. The CIBERSORT algorithm was utilized to evaluate the relative proportion of 22 immune cells in the two groups with the aid of R 4.1.2. Finally, with the help of the “reshape2” and “ggpubr”R package the immune score, stromal score, and ESTIMATE score were obtained.

### Prediction of immunotherapeutic sensitivity

The tumor immune exclusion score can be used to indicate how well HCC patients respond to immunotherapy. The immunophenoscore (IPS), which assesses the tumor immunogenicity determinants based on machine learning, is a biomarker for the response to immunotherapy. To reflect the response of different groups to immunotherapy, IPS were obtained from TCGA-LIHC.

### Single-cell analysis

We used Tumor Immune Single-cell Hub (TISCH) pipline (25) to characterize LIHC tumor microenvironment at single-cell resolution (http://tisch.comp-genomics.org/). A total of 1,944,551 cells from 76 datasets across 28 cancer types and 101,195 cells from 3 PBMC datasets are retained in TISCH database. In GSE125449 database, we used TISCH pipeline to annotate the cell types (cell-type annotations provided by the original studies, marker-based annotation method employed in MAESTRO using the DEGs between clusters, InferCNV method). Finally, we annotated eight different cell clusters, including fibroblasts, endothelial cells, exhausted CD8 T cells (CD8Tex), Plasma cells, B cells, malignant cells, Monocytes or Macrophages (Mono/Macro), hepatic progenitor. Gene expression was compared between different cells. In addition, due to the HAVCR1 was not annotated in GSE125449 dataset, we used CellMarker database to search the cell location (http://yikedaxue.slwshop.cn/search.php?quickSearchInfo=HAVCR1#framekuang).

### Cell culture

The Institute of Neuroscience, Soochow University, provided the L-O2 cell line and human hepatocellular carcinoma cell lines (Huh7, HepG2). In a humidified atmosphere (37°C with 5% CO2), L-O2 cells were cultured in RPMI1640 with 20% FBS, whereas Huh7 and HepG2 were cultured in DMEM with 10% FBS.

### Cell transfection

Cells were transfected using CP Reagent (Ribo-Bio, Guangzhou, China). HAVCR1 expression was knocked down using two different types of siRNAs. The following are the HAVCR1 siRNA sequences: si-HAVCR1#1: GACGGCCAATACCACTAAA, si-HAVCR1#2: CGACTGTTCTGACGACAAT. As a negative control group (si-con), nonspecific siRNA was used. After 48 hours of transfection, the cells were collected. The efficiency measurement was carried out by qRT-PCR and Western blot.

### Cellular function assays

Cell suspension (1000 cells/well) was inoculated in a DMEM medium containing 10% FBS in 96-well plates. We incubated the culture plates for 24, 48 and 72 hours at 37°C and 5% CO2. The CCK8 assay was used to assess cell viability. Optical densities of CCK8 were measured using a microplate reader at 450 nm. EdU and phagokinetic track motility assays has been described in detail in our previous article (26). Transwell chambers (24‐well, 12µm pore size, BIOFIL, China) were used to detect the migration of HuH7 and HepG2 after HAVCR1 silencing. The lower chamber was added with 0.6 mL DMEM with 20% FBS, whereas the upper chamber was added with around 7 × 10^4^ cells resuspended in Basic DMEM media and incubated overnight at 37°C with 5% CO_2_. Cells were fixed with 4% paraformaldehyde and stained with 2.5% crystal violet 24 hours later. Three microscopic views were selected randomly and counted by ImageJ. Transwell chambers (24‐well,12 µm pore size, BIOFIL, China) were also used in a cell invasion assay. The upper chamber was precoated with 250 µg/mL Matrigel (BD Bioscience) and was left uncoated for migration. Subsequent steps are similar to the “Transwell Assay”.

### qRT-PCR assay

HCC cells were seeded into 6-well plates at a density of 1×10^5^ cells in each well. To extract total RNA, lysis buffer was added to the culture. The QuantiTect Reverse Transcription Kit was then used to reverse transcribe the RNA into cDNA. qRT-PCR was performed through an SYBR Green PCR kit (Ribo-Bio, Guangzhou). The 2^-ΔΔCt^ method was used for the quantification of targeted mRNA. As an internal control, β-Actin mRNA was tested. **Table 2** lists the gene primer sequences in detail.

**Table 2:** Premier sequences for qRT-PCR analysis.

Premier	Sequences (5′–3′)
ANLN-F	TGCCAGGCGAGAGAATCTTC
ANLN-R	CGCTTAGCATGAGTCATAGACCT
UCK2-F	GCCCTTCCTTATAGGCGTCAG
UCK2-R	CTTCTGGCGATAGTCCTACTTC
LPCAT1-F	CGCCTCACTCGTCCTACTTC
LPCAT1-R	TTCCCCAGATCGGGATGTCTC
TTK-F	GTGGAGCAGTACCACTAGAAATG
TTK-R	CCCAAGTGAACCGGAAAATGA
KIF2C-F	CTCAGTTCGGAGGAAATCATGTC
KIF2C-R	TGCTCTTCGATAGGATCAGTCA
HAVCR1-F	TGGCAGATTCTGTAGGCTGGTT
HAVCR1-R	AGAGAACATGAGCCTCTATTCCA
MMP1-F	AAAATTACACGCCAGATTTGCC
MMP1-R	GGTGTGACATTACTCCAGAGTTG
CBX2-F	GCCCAGCACTGGACAGAAC
CBX2-R	CACTGTGACGGTGATGAGGTT
ACTB-F	TCAAGATCATTGCTCCTCCTGAG
ACTB-R	ACATCTGCTGGAAGGTGGACA

### Western blotting

In this procedure, 10% SDS–polyacrylamide gel electrophoresis (SDS-PAGE) was used to separate aliquots of 20 mg of protein from each treatment, which were then transferred to the polyvinylidene difluoride (PVDF) membrane (Millipore, Bedford, MA). After a 2-hour blocking procedure with 10% instant nonfat dry milk (BD, USA), membranes were incubated with specific antibodies overnight at 4°C followed by 100 minutes at 20°C with HRP-conjugated secondary antibodies. The next Western blotting protocols were reported previously (26). Data quantification was performed by ImageJ. The primary antibodies include anti-TTK (1:1000, BOSTER), anti-KIF2C (1:1000, BOSTER), anti-MMP1 (1:1000, BOSTER), anti-HAVCR1 (1:1000, BOSTER), anti-β-actin (1:2000, Abcam).

### Statistical analysis

All bioinformatics analyses were performed using the R platform (v.4.1.2). The data were presented as mean ± SD. One-way analysis of variance (ANOVA) was used, followed by Student’s t-test. Statistical significance was defined as a P-value of less than 0.05 (P < 0.05).

## Results

### Identification of FA metabolism-related DEGs between normal and HCC tissues

**Figure 1:**
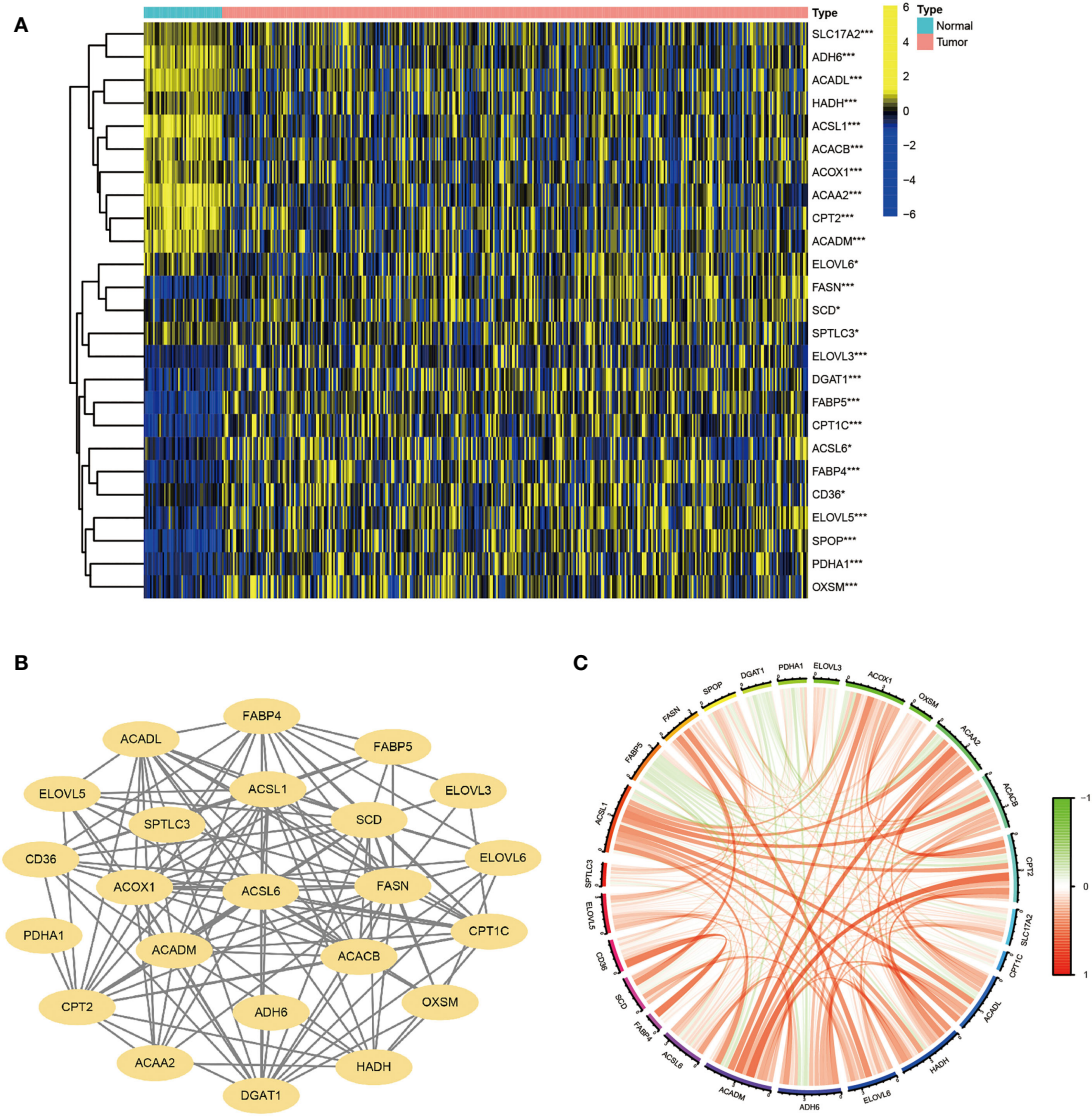
Identification of FA Metabolism-Related DEGs between Normal and HCC Tissues. The landscape of FA metabolism-related genes of HCC patients from TCGA database. **(A)** Heatmap showed DEG expression in two different tissues. **(B)** PPI network of the interactions. **(C)** The correlation network of these genes (*p < 0.05, **p < 0.01, ***p < 0.001).

A flow chart of the overall research is shown in **Figure S1**. 30 genes were chosen based on their roles in previous studies (27–33). The majority of FA metabolism-related genes were identified as DEGs (p < 0.05) using Heatmap analysis (**Figure 1A**). The protein-protein interaction (PPI) analysis was carried out on these DEGs using the Homo sapiens data set (with a confidence of 0.9) to better comprehend their interactions. The PPI network retained 23 hub DEGs that had complicated regulatory correlations (**Figure 1B**). Moreover, the correlation of these DEGs was analyzed and presented in **Figure 1C** (cutoff >0.4). We preliminarily concluded that the majority of these FA metabolism-related DEGs affect the tumorigenesis and tumor progression through mutual positive regulation.

### Clustering, construction of HCC classification according to genes associated with FA metabolism

**Figure 2:**
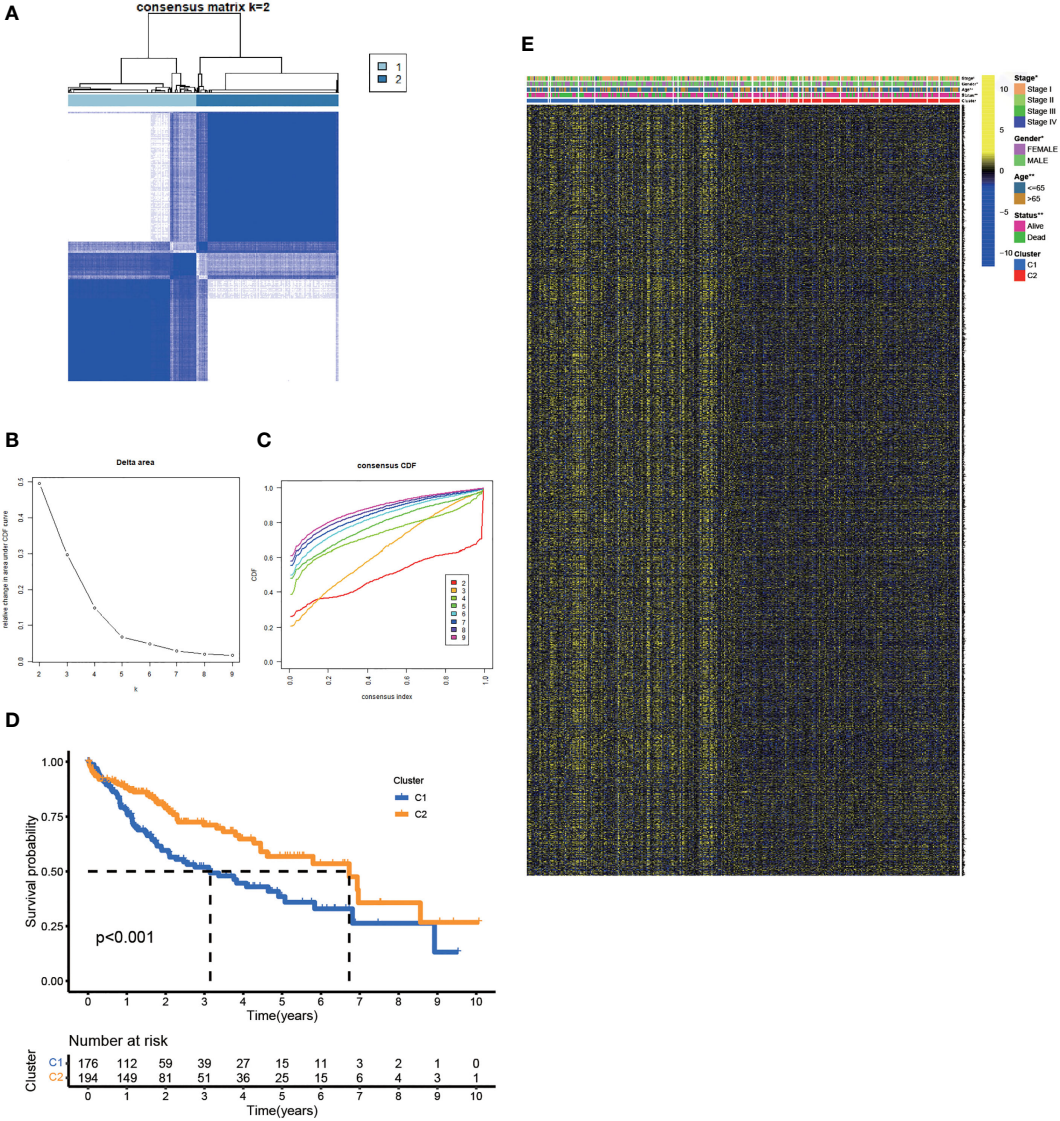
Clustering, Construction of HCC Classification According to Genes associated with FA Metabolism. **(A)** Patients were classified into two subtypes according to the consensus clustering matrix. **(B, C)** Consensus clustering model. **(D)** The KM analysis of the OS in the two subtypes. **(E)** Heatmap showed the correlation between the subtypes and clinicopathologic parameters (*p < 0.05, **p < 0.01, ***p < 0.001).

Based on the expression matrix of 30 FA metabolism-related DEGs, 2 clusters were identified using unsupervised clustering methods (k=2, **Figures 2A**–**C**). There were significant differences in OS time among them (**Figure 2D**). Thus, identifying prognostic genes related to FA metabolism was crucial. The DEGs between the two subtypes were then screened and obtained for subsequent analysis (|log2FC| > 1, p-value < 0.05). DEGs expression profiles and clinicopathologic parameters were shown on the heatmap. As expected, the expression of most DEGs, as well as the number of patients with stage III-IV, were significantly greater in cluster1 (**Figure 2E**).

### Functional analyses and the tumor immune microenvironment between FA metabolism-related subtypes

**Figure 3:**
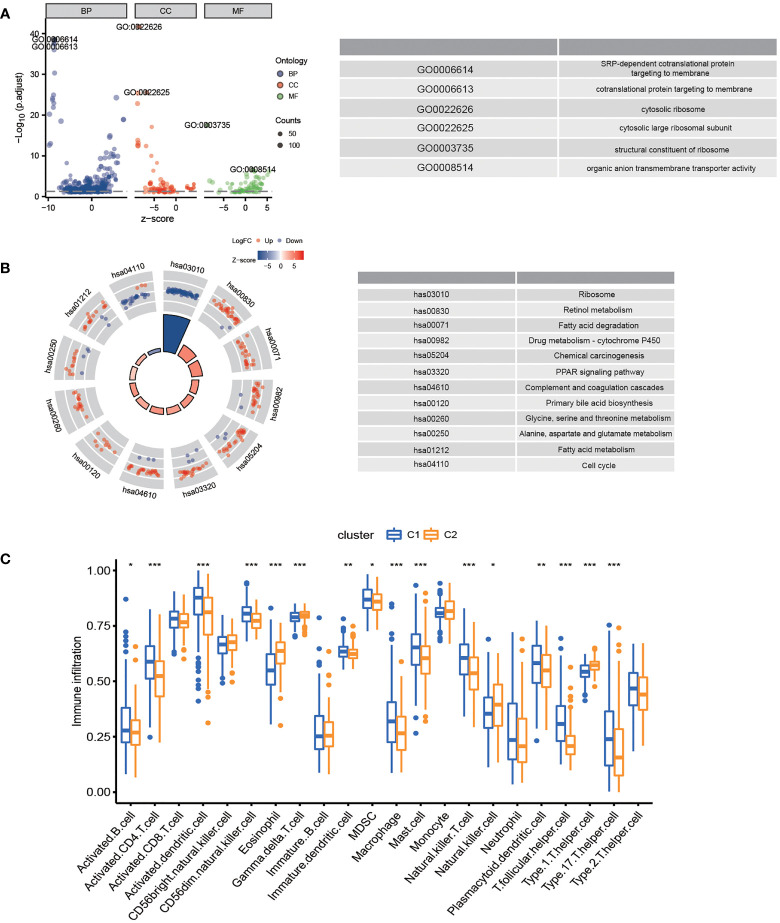
Functional Analyses and the Tumor Immune Microenvironment Between FA metabolism-related Subtypes. Potential biological pathways and tumor microenvironment affected by DEGs. GO **(A)** and KEGG **(B)** enrichment of DEGs. **(C)** Comparison of the ssGSEA scores between two subtypes (*p < 0.05, **p < 0.01, ***p < 0.001).

In the TCGA-LIHC cohort, DEGs between the two subtypes were analyzed to study the biological role and pathways of FA metabolism-related genes in more detail. As shown, GO analysis was divided into several parts: biological process (BP), cellular component (CC) and molecular function (MF) (**Figure 3A**). Notably, DEGs were also abundantly enriched in numerous immune responses, including neutrophil activation involved in immune response, neutrophil degranulation, T cell proliferation, mononuclear cell proliferation, regulation of leukocyte proliferation, lymphocyte apoptotic process, B cell apoptotic process, T-helper 1 cell differentiation (**Table 3**). Further, KEGG enrichment analyses indicated that the role of the Ribosome, Retinol metabolism, PPAR signaling pathway, Fatty acid metabolism, Cell cycle were enriched in both cohorts (**Figure 3B**). The tumor immune microenvironment (TIME) has profound implications for tumor diagnosis, patient survival outcomes, and sensitivity to clinical treatment (34). By analyzing the relationship between DEGs and TIME in the two subtypes, the potential immune mechanism of FA metabolism affecting the tumorigenesis of HCC was revealed. Results showed that both the subtypes were significantly associated with the immune scores. Extraordinarily, the patients with higher infiltration levels of immunosuppressive cells such as activated B cell, activated CD4 T cell, Mast cell and MDSC were more prone to cluster1 (**Figure 3C**). These findings confirmed that the expression of FA metabolism-related DEGs is associated with the prognosis and the TIME in HCC patients.

**Table 3:** Immunologic signature associated biological processes enriched in DEGs groups.

Gene set name	Size	Zscore	P.adjust	Qvalue
GO- neutrophil activation involved in immune response	82	-5.08	0.008	0.007
GO- neutrophil degranulation	81	-5.222	0.01	0.009
GO- T cell proliferation	37	-4.768	0.012	0.01
GO- mononuclear cell proliferation	50	-5.94	0.014	0.012
GO- regulation of leukocyte proliferation	41	-5.154	0.025	0.021
GO- lymphocyte apoptotic process	17	-3.638	0.028	0.023
GO- B cell apoptotic process	8	-2.828	0.048	0.041
GO- T-helper 1 cell differentiation	7	-1.89	0.046	0.039

### Establishment of a prognostic risk model in TCGA-LIHC cohort

**Figure 4:**
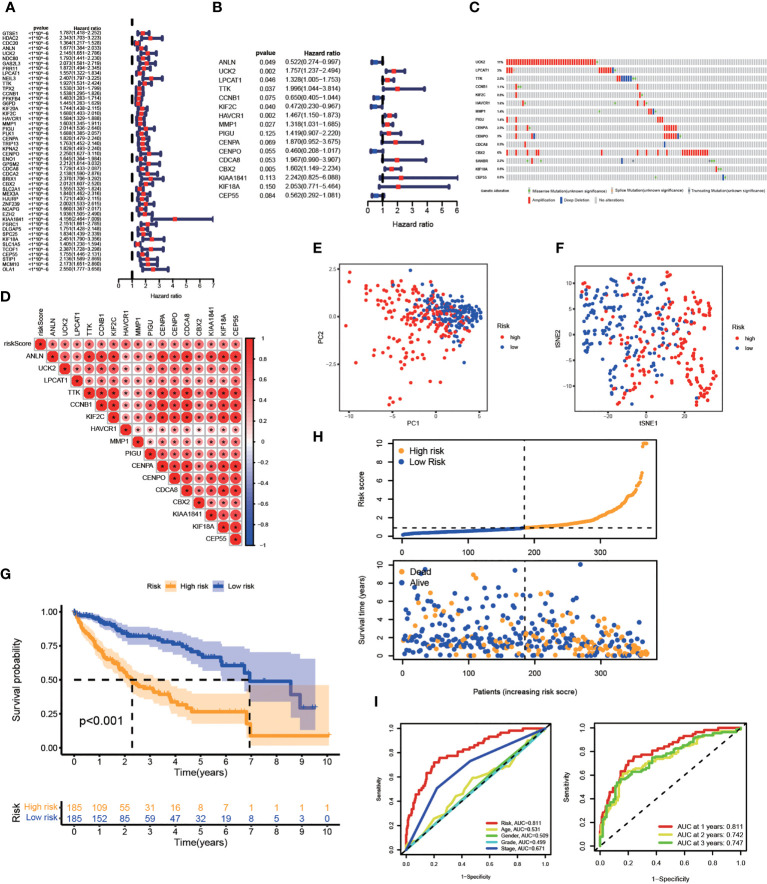
Establishment of A Prognostic Risk Model in TCGA-LIHC Cohort. Construction of a FA metabolism-related model in TCGA. **(A)** Univariate Cox regression analysis to find FA metabolism-related prognostic genes. **(B)** Multivariate Cox regression analysis to identify genes linked to the prognosis of FA metabolism. **(C)** The distribution of 16-gene genomic alterations in the TCGA-LIHC dataset. **(D)** Spearman correlation analysis of 16 genes. PCA analysis **(E)** and t-SNE analysis **(F)** based on risk scores. **(G)** The KM analysis of the OS based on risk scores. **(H)** Survival status distribution of these two groups. **(I)** ROC curve showing the accuracy of risk scores on the clinical parameters and year survival rate (*p < 0.05, **p < 0.01, ***p < 0.001).

The prognostic value of risk characteristics of FA metabolism was explored considering the complex regulation. To identify prognostic genes in the TCGA-LIHC, researchers used a univariate Cox regression analysis, which revealed that all genes were high-risk genes for HCC prognosis (**Figure 4A**). Multivariate Cox regression was used on the TCGA-LIHC cohort to further narrow down the potential gene range for developing a prognostic model. Genes including ANLN, UCK2, LPCAT1, TTK, CCNB1, KIF2C, HAVCR1, MMP1, PIGU, CENPA, CENPO, CDCA8, CBX2, KIAA1841, KIF18A, and CEP55, with their coefficients were subsequently maintained (**Figure 4B**). After exploring the prognosis of 16-gene, we used cBioPortal to analyze its mutation in HCC. As displayed in **Figure 4C**, all of these genes had great genetic variations, of which amplification was the most common variation characteristic. In addition, there was a significant positive correlation between these 16 genes (**Figure 4D**). As per the median risk score, we separated the patients into high- and low-risk groups, these two groups could be well-separated according to the PCA and t-SNE analysis (**Figures 4E**, **F**). According to the KM analysis, the association between high-risk score patients and poor prognosis was significant (**Figure 4G**). Furthermore, the number of mortalities showed an increasing trend with increasing risk scores (**Figure 4H**). The receiver operating characteristic (ROC) curve, also known as the sensitivity curve, was constructed to evaluate the model’s accuracy and feasibility in predicting patients’ survival (35), suggesting that the model exhibited a great predictive capability (AUC=0.811). Besides, the ROC curve also indicated the effectiveness of the FA metabolism-related signature in predicting the 1-, 3-, and 5-year survival rates in patients with HCC (**Figure 4I**).

### Association between risk genes and clinicopathologic parameters

Next, the association between the model and clinical parameters in HCC patients was explored. We discovered that these risk genes were substantially expressed in the high-risk group, as shown by the heatmap that shows risk gene expression profiles and clinicopathologic parameters. Moreover, there were significant differences between tumor stage and grade (**Figure S2A**). Notably, patients having higher risk scores may be in higher stages, whether AJCC stage or T stage (**Figure S2B**). Then, we divided the patients into several subgroups based on different clinical parameters such as gender (female *vs* male), age (> 65 *vs* ≤ 65), AJCC stage (I-II *vs* III-IV), and T stage (T1 *vs* T2-4). The KM analysis revealed that the high-risk patients had a lowered survival rate in all conditions (**Figures S2C–F**). Overall, the model constructed is highly correlated with clinicopathologic parameters and can guide the prognosis of HCC patients.

### Independent prognostic value of the model

**Figure 5:**
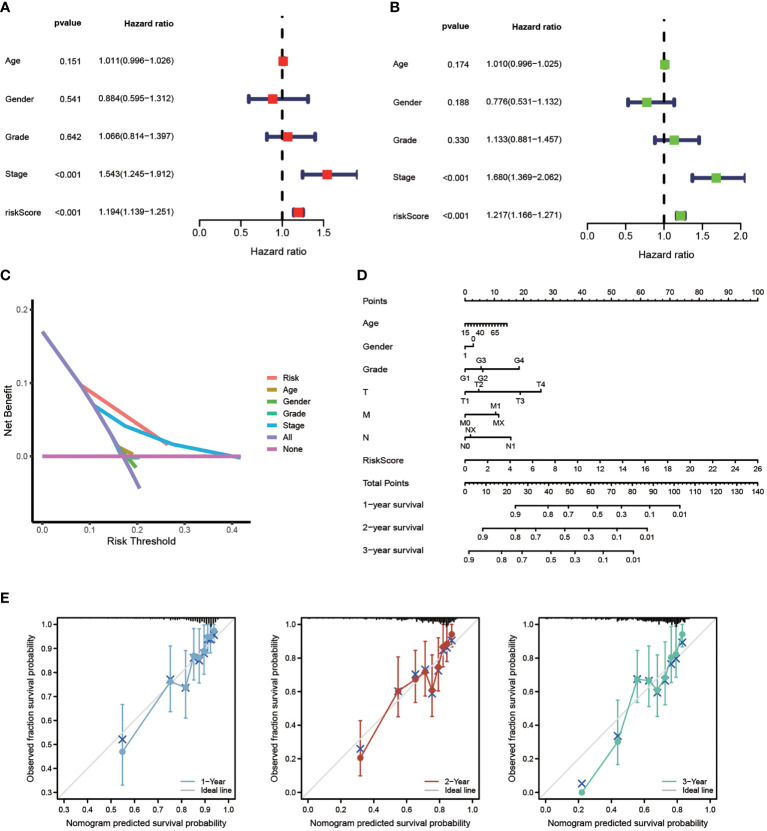
Independent Prognostic Value of The Model. Univariate Cox **(A)** and Multivariate Cox **(B)** analysis to assess the independence of the model. **(C)** DCA was performed to present the net benefit of risk score compared to clinical parameters. **(D)** Nomogram survival prediction of HCC patients with risk score. **(E)** Calibration plot of the nomogram (*p < 0.05, **p < 0.01, ***p < 0.001).

The efficiency of the model was tested in the TCGA-LIHC cohort. Based on univariate COX analysis, a high-risk score was shown to be correlated with poor prognosis significantly (p < 0.001, HR = 1.194, 95% CI: 1.139 - 1.251). The other variable associated with a worse prognosis was a stage (**Figure 5A**). According to multivariate Cox analysis, a higher risk score was revealed to be independently associated with poorer survival, supporting its potential for being an independent prognostic factor for HCC (p < 0.001, HR = 1.217, 95% CI: 1.166 - 1.217) (**Figure 5B**). Notably, DCA, a novel method that is used to assess clinical predictive models, diagnostic tests, and molecular markers (36), showed that our risk model achieves greater net benefit than any one single independent clinical parameter (**Figure 5C**). Additionally, the nomogram (C-index > 0.7) based on the clinical parameters and risk scores could effectively predict the probability of the1-, 2-, and 3- years OS (**Figure 5D**). Calibration curve results verified high agreement between nomogram predictions and actual observations (**Figure 5E**).

### Validation of the risk signature in ICGC-LIRI cohort

To further verify the model’s predictive accuracy, 231 HCC cases were extracted from the ICGC database to establish a test cohort. In the LIRI-JP cohort, 16 risk genes were all up-regulated, as shown in **Figure S3A**. Then, using these risk genes to separate the LIRI-JP cohort into two groups, we observed that patients in the high-risk group had higher mortality and shorter survival periods (**Figure S3B**). Based on the KM analysis, the OS of patients belonging to the low-risk group was higher (**Figure S3C**) 1-, 3-, and 5-year AUC values were 0.619, 0.595, and 0.950, respectively (**Figure S3D**), and the PCA plot validated that the high- and low-risk groups could be separated (**Figure S3E**). The association of high- and low-risk scores and OS in HCC patients was further validated in various clinical parameter subgroups. Based on the KM analysis, patients with high-risk scores had a lower survival rate when they were female, over 65, in stages I-II, and had primary malignancy (**Figure S3F**). All these suggest the reliability of the model.

### The potential molecular mechanism of the model

GSEA was applied to analyze the transcript message of HCC patients. Interestingly, the activity of metabolic pathways such as cytochrome P450 drug metabolism, FA metabolism, and retinol metabolism, were shown to be enriched in the low-risk group, according to KEGG enrichment analysis (**Figure S4A**). Cell cycle, DNA replication, ECM receptor interaction, neuroactive ligand-receptor interaction, and oocyte meiosis were enriched in the high-risk group (**Figure S4B**). Then, we performed GSVA enrichment to further explore potential signaling pathways. As shown in **Figure S4C**, these model genes were significantly enriched in most signaling oncogenic pathways and were positively correlated, including WNT, VEGF, Notch, and mTOR signaling pathways. In some pathways, such as PPAR and ADIPOCYTOKINE signaling pathways, these genes show consistent negative correlations. In addition, immune-related pathways, such as T cell receptor signaling pathway, B cell receptor signaling pathway were also enriched. These results provided a novel strategy for our subsequent research to find potential therapeutic targets.

### TME infiltration and immunotherapy

**Figure 6:**
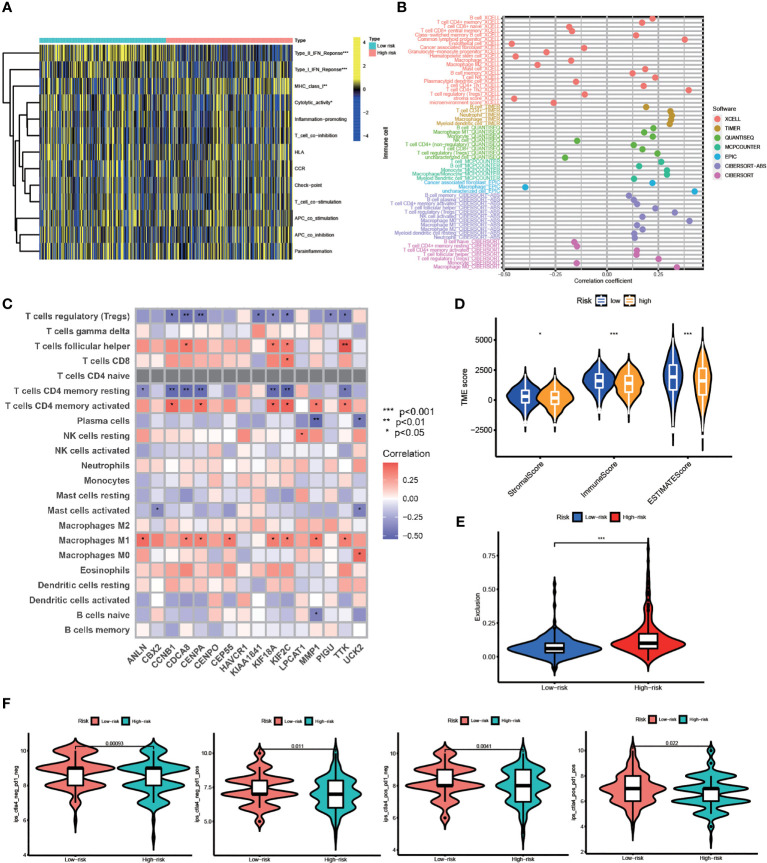
TME Infiltration and Immunotherapy. **(A)** Heatmap of the distribution of 13 types of immune function pathways between two groups. **(B)** Immune cell infiltration analysis based on different algorithms. **(C)** Correlation between immune-related cells and 16 genes. **(D)** TME score, including Stromal score, Immune score and Estimates score. **(E)** Exclusion score calculated by TIDE algorithm. **(F)** Immunotherapy score between two groups (po, positive; neg, negative) (*p < 0.05, **p < 0.01, ***p < 0.001).

Based on our findings above, we suggested this prognostic model is closely correlated with immune infiltration. Using ssGSEA, we systematically evaluated 13 types of immune function pathways to further assess the immune status-related association between the two groups. The risk score was highly associated with Type II IFN Response, Type I IFN Response, MHC class I, and Cytolytic activity, according to Heatmap analysis results (**Figure 6A**). The distribution of immune cells calculated by XCELL, TIMER, QUANTISEQ, MCPCOUNTER, EPIC, and CIBERSORT was also explored. HCC patients belonging to the high-risk group had higher proportions of immune cells including B Cell, T Cell, Macrophage, and so on (**Figure 6B**). The correlation between these 16 risk genes and immune cells was also performed by the CIBERSORT algorithm and presented in **Figure 6C**. The stromal score, immune score, and estimate score were then generated using the ESTIMATE algorithm. Furthermore, low-risk group patients had a higher stromal score (p < 0.05), immune score (p < 0.001) and estimate score (p < 0.001) (**Figure 6D**). Immunotherapy is a new type of therapy for a variety of cancers, including HCC. Regarding the response to immunotherapy in these two groups of HCC patients, we found a higher Exclusion score in the high-risk group, indicating a worse effect on receiving immunotherapy in the high-risk group (**Figure 6E**). Anti-PD-1/PD-L1 therapies have emerged as an effective treatment option, especially in HCC (37). Significant differences in immunotherapy scoring mechanisms were revealed in all four immunotherapy regimens; a lower risk score suggests a greater anti- PD-1/L1 therapeutic efficacy (**Figure 6F**). FA metabolism-related prognostic model was associated with anti-PD-1/L1 immunotherapy, as expected, and can potentially predict immunotherapy response.

### Differential expression of independent prognostic genes and validation

**Figure 7:**
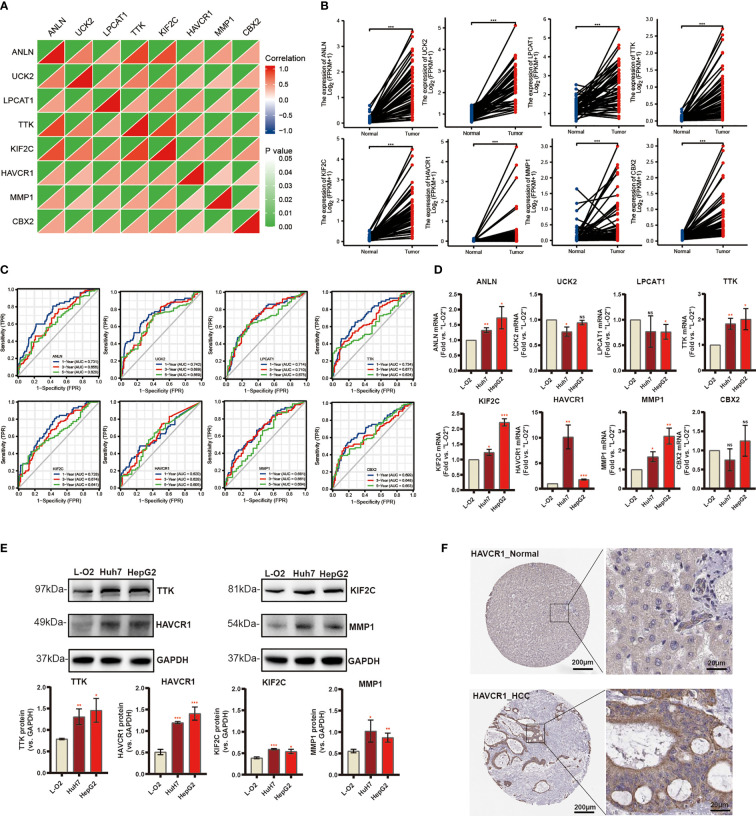
Differential Expression of Independent Prognostic Genes and Validation. **(A)** Spearman correlation analysis of eight candidate genes in the TCGA-LIHC cohort. **(B)** Eight risk genes expression in paired tissues from TCGA database. **(C)** The ROC curve of independent risk genes in TCGA-LIHC cohort. **(D)** The mRNA levels of quantified using qRT-PCR analysis in human liver cell line and two HCC cell lines. **(E)** The protein expression of TTK, KIF2C, HAVCR1, and MMP1. **(F)** Immunohistochemistry of the HAVCR1 from the HPA database (*p < 0.05, **p < 0.01, ***p < 0.001).

Subsequently, eight candidate genes including ANLN, UCK2, LPCAT1, TTK, KIF2C, HAVCR1, MMP1, and CBX2 were selected based on multivariate Cox regression analyses (p < 0.05). Notably, the correlation between these genes was significantly positive (**Figure 7A**). We further explored the expression of these prognostic genes in 50 pairs of samples from the TCGA-LIGC cohort, and the results revealed that significant elevation of the mRNA expression levels of these genes in HCC tissue (**Figure 7B**). In addition, the ROC curve displays a favorable predictive value of these independent genes over 1, 3, and 5 years (**Figure 7C**). To verify mRNA expression in HCC, we performed qRT-PCR in human liver epithelial (LO2) and two HCC cell lines (Huh7, HepG2) (**Figure 7D**). To test TTK, KIF2C, HAVCR1, and MMP1 protein expressions, Western blotting assays were then performed and the results confirmed the up-regulation of all these proteins in HCC cell lines (**Figure 7E**). Extraordinarily, we found dramatically increased HAVCR1 mRNA and protein expression in HCC cell lines. The protein levels of HAVCR1 were validated by the HPA database (**Figure 7F**).

### Single cell analysis

In GSE125449 database, we used TISCH pipeline to annotate the cell types (cell-type annotations provided by the original studies, marker-based annotation method employed in MAESTRO using the DEGs between clusters, InferCNV method). Finally, we annotated eight different cell clusters (**Figure S5A**), including fibroblasts, endothelial cells, exhausted CD8 T cells (CD8Tex), plasma cells, B cells, malignant cells, monocytes or macrophages (mono/macro), hepatic progenitor. Subsequently, we explored the expression of our risk genes in different cell types. Unfortunately, the HAVCR1 was not annotated in this dataset. Hence, we used CellMarker database to search the cellular location, and we found HAVCR1 was mainly expressed in normal liver cells. Moreover, the TISCH results showed that most of the genes were not significantly expressed in B cells, and only LPCAT1 and CBX2 were significantly expressed in B cells (**Figure S5B**). Among them, the ANLN, UCK2, KIF2A were more evenly distributed in cell types other than B cells. TTK was significantly expressed in CD8Tex cells, hepatic progenitor, malignant, and mono/macro cells. Finally, MMP1 was only significantly expressed in endothelial cells, fibroblasts, hepatic progenitor and malignant cells. Taken together, our data showed risk genes were not only expressed in malignant cells, and different genes had expression heterogeneity.

### HAVCR1 silencing inhibits HCC cell proliferation, motility, and invasion

**Figure 8:**
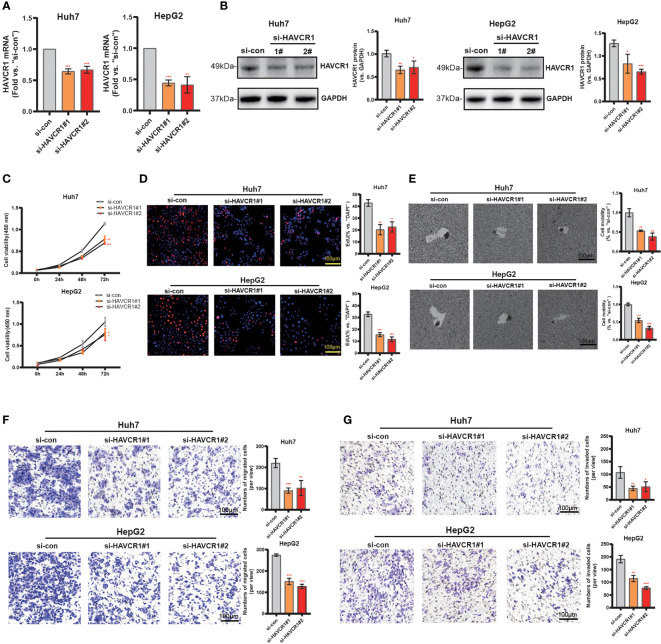
HAVCR1 Silencing Inhibits HCC Cell Proliferation, Migration, and Invasion. Established human HCC cell lines (HuH7 and HepG2) **(A–F)**, bearing the HAVCR1 siRNA (“si-HAVCR1#1” and “si-HAVCR1#2”). qRT-PCR **(A)** and Western blotting **(B)** were employed for assessing the HAVCR1 mRNA and protein expression after si-HAVCR transfection. CCK-8 **(C)** and EdU assay **(D)** were used to test the proliferation of HuH7 and HepG2 cells. Phagokinetic track motility assay **(E)** was used measure the motility of cells. Cell migration and invasion were measured by “Transwell” **(F)** and “Matrigel Transwell” assays **(G)** (*p < 0.05, **p < 0.01, ***p < 0.001).

Considering the model was strongly associated with the HCC, the independent prognostic genes may have a greater impact on the biological function of HCC cells. We selected HAVCR1 with the largest expression difference to further verify our hypothesis. The relationship between HAVCR1 expression and prognosis of HCC was validated in ICGG database (**Figure S6**). Moreover, HAVCR1 expression was positively correlated with histologic grade (**Table 4**). HAVCR1 siRNA was transduced to Huh7 cells and HepG2 cells. Robust decrease of HAVCR1 mRNA and protein levels by HAVCR1 siRNA was confirmed by qRT-PCR and Western Blotting assay (**Figures 8A**, **B**). CCK-8 assay results showed a significant reduction in viability by HAVCR1 siRNA in HuH7 and HepG2 cells (**Figure 8C**). Huh7 and HepG2 cell proliferation was largely inhibited by decreased EdU-positive nuclei ratio after siRNA-mediated knockdown of HAVCR1 (**Figure 8D**). In addition, the phagokinetic track motility assay results confirmed that cell motility was significantly inhibited by siRNA (**Figure 8E**). Moreover, using the “Transwell” assay, it was shown that HCC cell migration was attenuated (**Figure 8F**). A significant decrease in the HCC cell invasion was also shown by “Matrigel Transwell” assays (**Figure 8G**). These findings implied that HAVCR1 siRNA can inhibit the biological function in HCC, but the detailed mechanism needs to be further illuminated.

**Table 4:** Association of HAVCR1 expression with clinicopathological parameters in TCGA-LIHC.

Characteristic	Low expression of HAVCR1	High expression of HAVCR1	p
n	187	187	
Age, n (%)			0.643
<=60	86 (23.1%)	91 (24.4%)	
>60	101 (27.1%)	95 (25.5%)	
Gender, n (%)			0.185
Female	54 (14.4%)	67 (17.9%)	
Male	133 (35.6%)	120 (32.1%)	
Pathologic stage, n (%)			0.436
Stage I	89 (25.4%)	84 (24%)	
Stage II	44 (12.6%)	43 (12.3%)	
Stage III	38 (10.9%)	47 (13.4%)	
Stage IV	1 (0.3%)	4 (1.1%)	
AFP(ng/ml), n (%)			< 0.001***
<=400	122 (43.6%)	93 (33.2%)	
>400	19 (6.8%)	46 (16.4%)	
Histologic grade, n (%)			0.003**
G1	38 (10.3%)	17 (4.6%)	
G2	90 (24.4%)	88 (23.8%)	
G3	52 (14.1%)	72 (19.5%)	
G4	3 (0.8%)	9 (2.4%)	
Age, median (IQR)	61 (52.5, 69)	61 (51, 68)	0.290

## Discussion

Recently, the HCC incidence has been rising (38). Despite the advances in cancer prevention, early screening, and current treatment options, the prognosis for HCC is extremely poor (39). The current diagnostic options are not sensitive and accurate enough (40). Thus, it is extremely necessary to explore novel and efficient markers of diagnosis and prognosis for improving the OS of HCC patients (41). Increasing studies have shown that metabolic dysregulation is one of the main characteristics of malignant tumor cells, leading to growth, angiogenesis, proliferation, and invasion (42–44). FA metabolism, as an important part of energy metabolism, is involved in multiple biological processes for promoting tumorigenesis and progression (13). FA metabolism has been shown to play a key role in the onset and progression of HCC (39). Most research focused only on a single regulatory factor of FA metabolism in HCC (10, 29, 45), however, integrated models of multiple important genes involved in FA metabolism are needed. He et al. had explored FA metabolism-related risk genes in HCC by constructing a prognostic model but failed to further explore the role of these genes in the onset and progression of HCC (46). Identification of key molecular markers associated with FA metabolism and clarification of their roles in the progression of HCC is necessary.

Our present study first systematically investigated 30 genes highly associated with FA metabolism in patients with HCC and revealed that 14 genes among them were significantly upregulated, while 10 genes were downregulated. Most of these genes were positively correlated. Then, we identified two clusters based on these FA metabolism-related genes by performing consensus clustering, these two clusters showed significant survival differences. DEGs were then compared between the two clusters, with the results revealing enrichment of DEGs in immune processes. Currently, the investigation of FA metabolism-related genes in the TIME in HCC is insufficient. Our study showed that patients with few immunosuppressive cells favored cluster 2 compared to those with more immunosuppressive cells. High infiltration of immunosuppressive cells suggested the tumor microenvironment was inhibited, leading to the poor prognosis of HCC. As the consensus clustering was based on 30 FA metabolism-related genes expression, we inferred that FA metabolism was closely related to the prognosis and TIME of HCC patients.

DEGs were analyzed using univariate and multivariate Cox regression analyses to develop a 16-gene risk model to further investigate the prognostic significance of FA metabolism in HCC. The model possessed great predictive accuracy and could guide the prognosis among the patients with different clinical parameters. These results were validated in the ICGC external validation dataset. GSEA enrichment was performed to further explore potential molecular mechanism of the model, several pathways (e.g., Cell cycle, DNA replication, ECM receptor interaction, and so on) involving the tumorigenesis were enriched in the high-risk group. It is widely recognized that abnormal cell cycle and DNA replication was considered a biomarker of HCC (47, 48). GSVA enrichment showed the model genes were significantly enriched in most signaling oncogenic pathways and were positively correlated, suggesting these risk genes play the oncogenic role in HCC. All these findings provide ideas for our future mechanism research.

Robust evidence has shown an intimate relationship between FA metabolism and tumor immunity (49, 50). The immune infiltration status of 22 immune cells was analyzed by the ssGSEA algorithm, revealing that several immune cells, including B Cell, T Cell, and macrophage were associated with the risk score significantly. Subsequently, the risk score was highly associated with Type I and II IFN Response. The activation of IFN-I on liver cells controls glucose homeostasis and lipid metabolism which supports cell proliferation and tumorigenesis (51). Interestingly, the correlation between IRF-1, IRF-2, and PD-L1 was significantly positive. Overexpression of IRF-2 could down-regulate PD-L1 promoter activity and protein levels which was induced by IFN-γ (52). Anti-PD-1/PD-L1 therapy has improved outcomes in a range of advanced malignancies, including HCC, since its discovery. It is worth noting that although immunotherapy has many advantages in anti-cancer treatment, its efficacy shows strong individual variability (53). Our findings revealed a significant correlation between risk score and immunotherapy efficacy. A low-risk score indicates a better therapeutic effect of anti-PD-1/PD-L1 therapies. The prognostic model could effectively predict the suitability of HCC patients for anti-PD1/PDL1 immunotherapy, further supporting that FA metabolism is indispensable in shaping individual TIME characterizations.

Next, we chose eight risk genes including ANLN, UCK2, LPCAT1, TTK, KIF2C, HAVCR1, MMP1, and CBX2 based on multivariate Cox regression analyses above (p < 0.05). These eight genes RNA-seq paired sample data obtained from TCGA, showed higher mRNA levels in the HCC tissues compared to the normal tissues, and the ROC curve of these independent genes indicated a favorable predictive value over 1, 3, and 5 years. Later, qRT-PCR and Western blotting assays showed that mRNA and protein expression of TTK, KIF2C, HAVCR1, and MMP1 mRNA between tumors and normal tissues are significantly different. Threonine and tyrosine protein kinase (TTK), which is also known as monopolar spindle 1 (Mps1), acts as an oncogenic gene in a variety of cancers (54, 55). Previous studies indicate that KIF2C promotes the growth, invasion, and metastasis of HCC by mediating the Ras/MAPK signaling pathway (56). MMP1, which is an interstitial collagenase, has been implicated in the proliferation and metastasis in a variety of malignancies (57–59). HAVCR1 also known as T-cell immunoglobulin mucin domains (TIM)-1, is overexpressed in renal cell carcinoma (60), human colorectal cancer (61), and gastric adenocarcinomas (62), promoting the occurrence and progression of tumors. Mori ever reported HAVCR1 could mediate FA uptake to promote progress of kidney disease (63). Here, we focused on the HAVCR1, which shows the most significant difference in expression between normal liver cells and HCC cell lines. Functional experiments found that siRNA-induced HAVCR1 silencing robustly inhibited HCC cell growth, proliferation, migration, and invasion, suggesting that HAVCR1 could play an oncogenic role in HCC, however, the detailed underlying molecular mechanisms need to be further elucidated.

There are some unique superiorities in our research. Specifically, our study systematically evaluated the expression and prognostic value of FA metabolism-related genes in HCC. A better prognostic model consisting of 16 genes was established and further validated in the ICGC dataset. We found that HCC patients with high-risk scores had significantly poor prognoses and were highly correlated with clinicopathologic parameters. Notably, our model predicts patient survival with higher accuracy than previous models. The model genes were significantly enriched in most signaling oncogenic pathways and were significantly associated with tumor immunity and can predict the efficacy of immunotherapy. In addition, we studied the effect of HAVCR1 on the biological functions of HCC cells, finding that HAVCR1 silencing inhibits HCC cell growth, proliferation, and motility.

Exploring the prognostic value of FA metabolism-related genes also lays a foundation for our future mechanism research. Nevertheless, there are some limitations of our study that must be considered. Firstly, all analyses were performed using TCGA and ICGC databases, and more clinical patient data is needed to confirm its accuracy. Secondly, further experiments are required to investigate the correlation between our prognostic model and the tumor microenvironment. Finally, we only have preliminarily explored the effects of HAVCR1 on HCC cell functions by siRNA silencing, more genetic modifications need to be performed to further confirm the role of HAVCR1 on HCC cells, and the underlying molecular mechanisms need to be further elucidated.

## Conclusion

We constructed a FA metabolism-related prognostic model of genes that possessed predictive accuracy based on the data of the LIHC cohort available in the TCGA database. The prognostic model was also significantly associated with tumor immunity and can predict the efficacy of anti-PD-1 immunotherapy. HAVCR1, a gene highly related to FA metabolism, has been proven to promote the growth, proliferation, migration, and invasion of HCC cells. The FA metabolism-related signatures could provide further possibilities to predict the progression and prognosis. Our study provides a novel idea for future research on personalized treatment strategies for HCC patients.

## Data availability statement

The original contributions presented in the study are included in the article/**Supplementary Material**. Further inquiries can be directed to the corresponding author/s.

## Ethics statement

This study was approved by Ethics Committee of Affiliated Kunshan Hospital of Jiangsu University.

## Author contributions

X-RZ and J-QZ performed formal analysis and drafted the manuscript. Y-FC is responsible for project management. J-JL and JS participated in software and data analysis. Y-YL and S-QP conducted the experiment. Article was written, edited, and reviewed by X-RZ and Y-YL. M-BC and Y-PD guided and revised the manuscript. All authors read and approved the final submission. X-RZ, J-QZ and Y-FC contributed equally to this article.

## Funding

This project was supported by grants from the National Natural Science Foundation of China (82072712) and Suzhou Science and Technology plan project (KJXW2019064).

## Acknowledgments

We thank all our authors listed in this manuscript.

## Conflict of interest

The authors declare that the research was conducted in the absence of any commercial or financial relationships that could be construed as a potential conflict of interest.

## Publisher’s note

All claims expressed in this article are solely those of the authors and do not necessarily represent those of their affiliated organizations, or those of the publisher, the editors and the reviewers. Any product that may be evaluated in this article, or claim that may be made by its manufacturer, is not guaranteed or endorsed by the publisher.

## Supplementary material

Supplementary Figure 1 Overall design of the study.

Supplementary Figure 2 Association Between Risk Genes and Clinicopathologic Parameters. **(A)** Heatmap indicating the links between clinicopathological parameters and different risk groups. PCA analysis. **(B)** Risk scores are classified by cluster, gender, stage and T stage. KM analysis of the OS based on gender **(C)**, age **(D)**, Stage **(E)**, T stage **(F)** (*p < 0.05, **p < 0.01, ***p < 0.001).

Supplementary Figure 3 Validation of The Risk Signature in ICGC-LIRI Cohort. **(A)** The volcano plot shows 16 risk genes in the ICGC-LIRI cohort. **(B)** Survival status distribution in ICGC-LIRI cohort. **(C)** KM analysis of the OS based on risk scores in the ICGC-LIRI cohort. The ROC curve **(D)** and PCA analysis **(E)** in the ICGC-LIRI cohort. (F) KM analyses based on different clinical parameters (*p < 0.05, **p < 0.01, ***p < 0.001).

Supplementary Figure 4 The Potential Molecular Mechanism of the Model. GSEA in low-risk groups **(A)** and high-risk groups **(B)**. **(C)** GVSA of 16 risk genes (*p < 0.05, **p < 0.01, ***p < 0.001).

Supplementary Figure 5 Single cell analysis of different genes expression.

Supplementary Figure 6 KM analysis comparing the high and low expression of HAVCR1 in HCC in the ICGC-LIRI cohort.

## Abbreviations

HCC, hepatocellular carcinoma; FA, fatty acid; TCGA, The Cancer Genome Atlas; ICGC, The International Cancer Genome Consortium; DEGs, differentially expressed genes; PPI, protein-protein interaction network; NES, normalized enrichment score; GO, Gene ontology; KEGG, Kyoto Encyclopedia of Genes and Genomes; OS, overall survival; ROC, receiver operating characteristic; DCA, decision curve analysis; GSEA, gene-set enrichment analysis; GSVA, gene-set variation analysis; TME, Tumor Microenvironment; TIME, Tumor Immune Microenvironment; PD1, programmed cell death protein 1; PD-L1, Programmed death-ligand 1; HR, Hazard ratio.
