# Supplementary figures and images for: Bioinformatics analysis and experimental verification of the prognostic and biological significance mediated by fatty acid metabolism related genes for hepatocellular carcinoma
Source: Front Oncol. 2022 Aug 2;12:972744. doi: 10.3389/fonc.2022.972744 (PMC9378871; doi:10.3389/fonc.2022.972744)

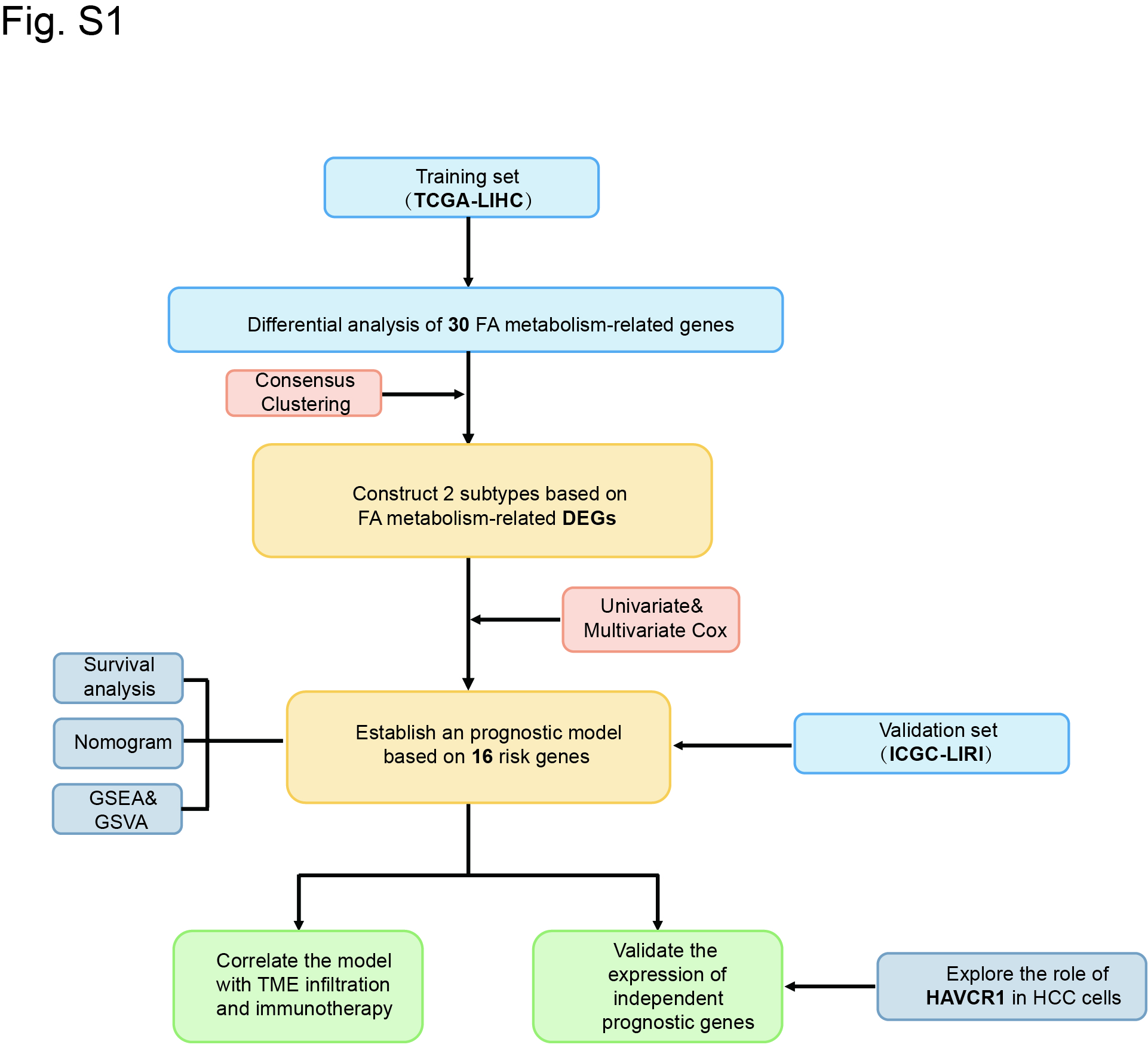

Supplement: Supplementary Figure 1 — Overall design of the study. [file Image_1.jpeg]

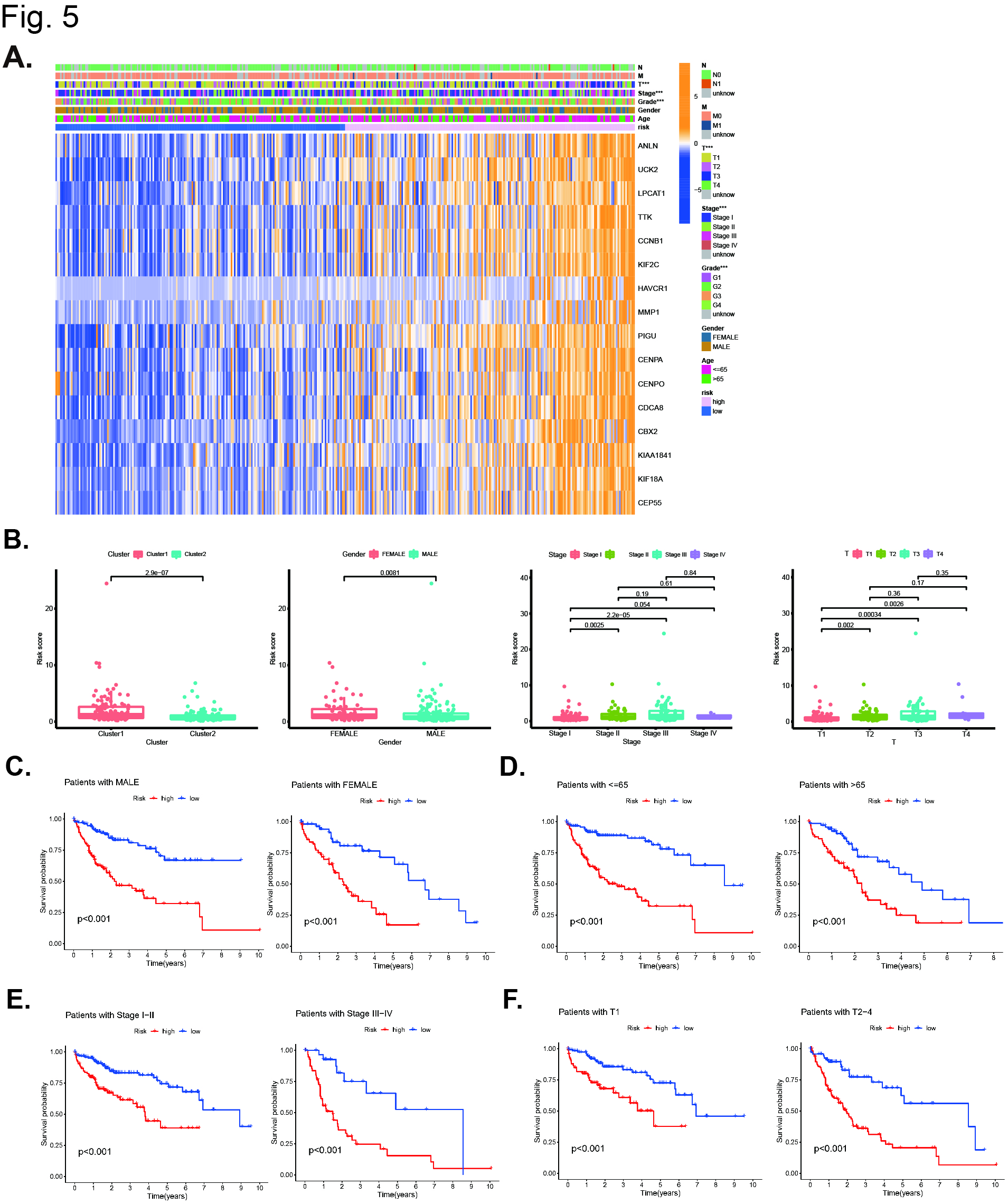

Supplement: Supplementary Figure 2 — Association Between Risk Genes and Clinicopathologic Parameters. (A) Heatmap indicating the links between clinicopathological parameters and different risk groups. PCA analysis. (B) Risk scores are classified by cluster, gender, stage and T stage. KM analysis of the OS based on gender (C), age (D), Stage (E), T stage (F) (*p < 0.05, **p < 0.01, ***p < 0.001). [file Image_2.jpeg]

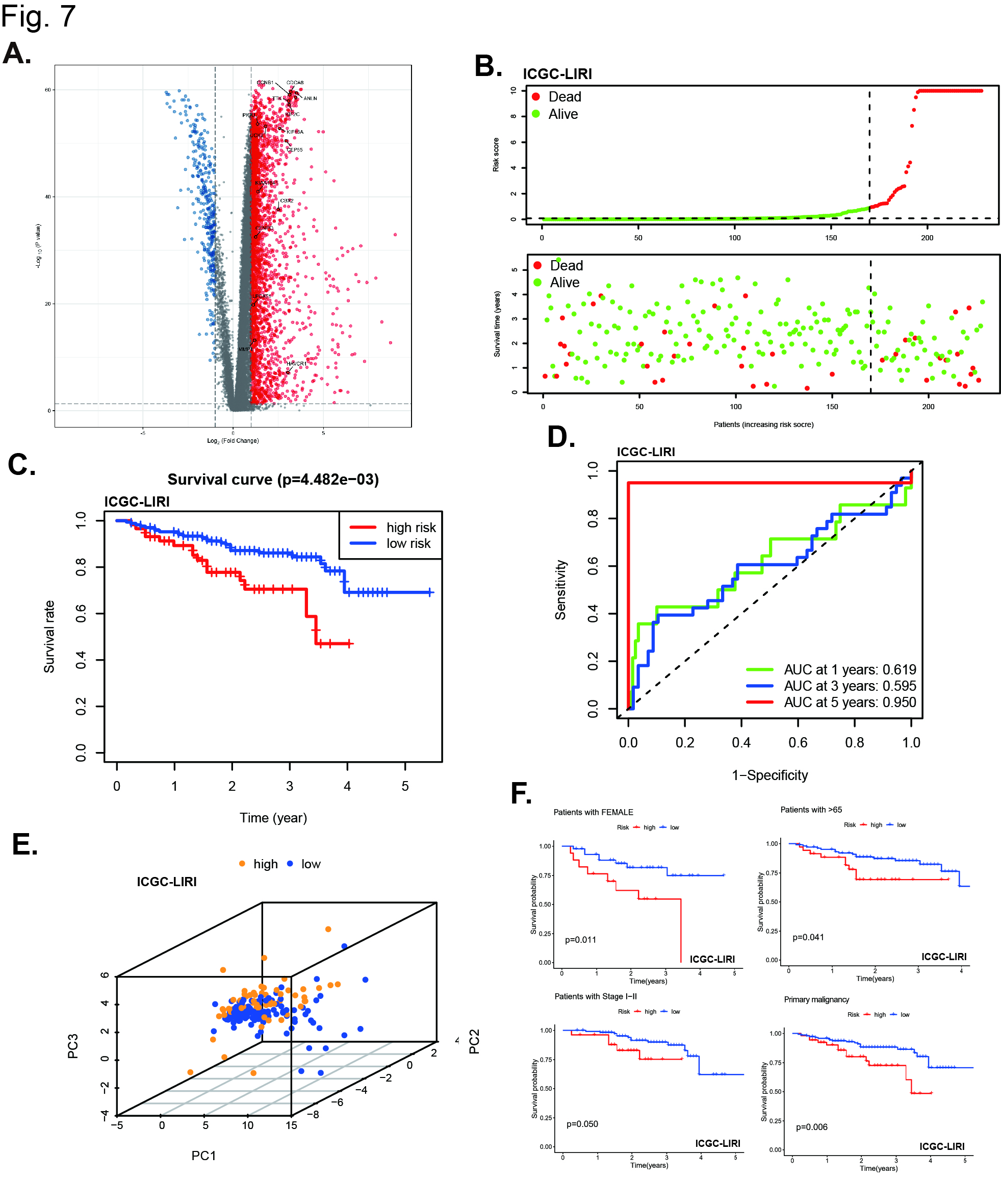

Supplement: Supplementary Figure 3 — Validation of The Risk Signature in ICGC-LIRI Cohort. (A) The volcano plot shows 16 risk genes in the ICGC-LIRI cohort. (B) Survival status distribution in ICGC-LIRI cohort. (C) KM analysis of the OS based on risk scores in the ICGC-LIRI cohort. The ROC curve (D) and PCA analysis (E) in the ICGC-LIRI cohort. (F) KM analyses based on different clinical parameters (*p < 0.05, **p < 0.01, ***p < 0.001). [file Image_3.jpeg]

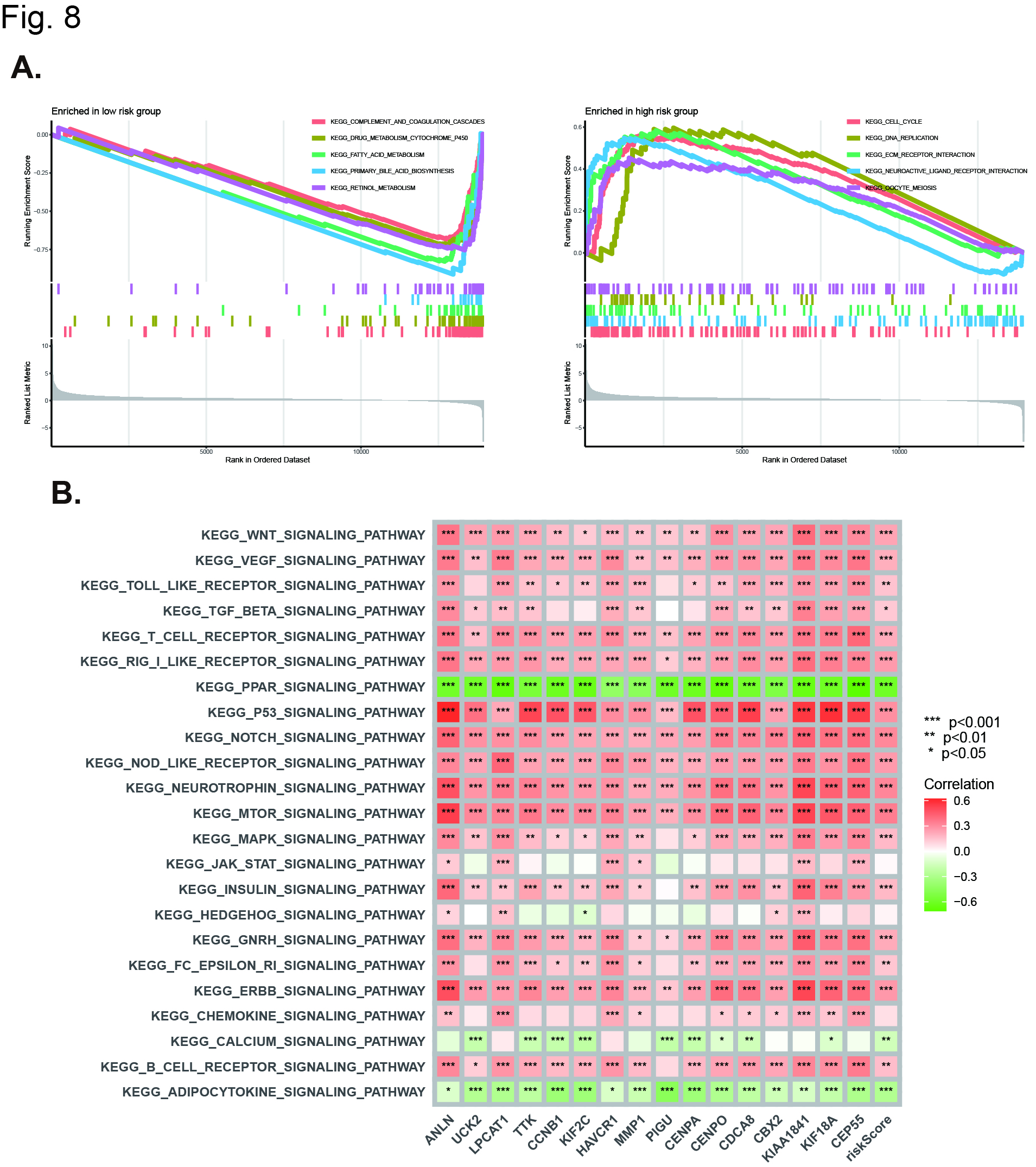

Supplement: Supplementary Figure 4 — The Potential Molecular Mechanism of the Model. GSEA in low-risk groups (A) and high-risk groups (B). (C) GVSA of 16 risk genes (*p < 0.05, **p < 0.01, ***p < 0.001). [file Image_4.jpeg]

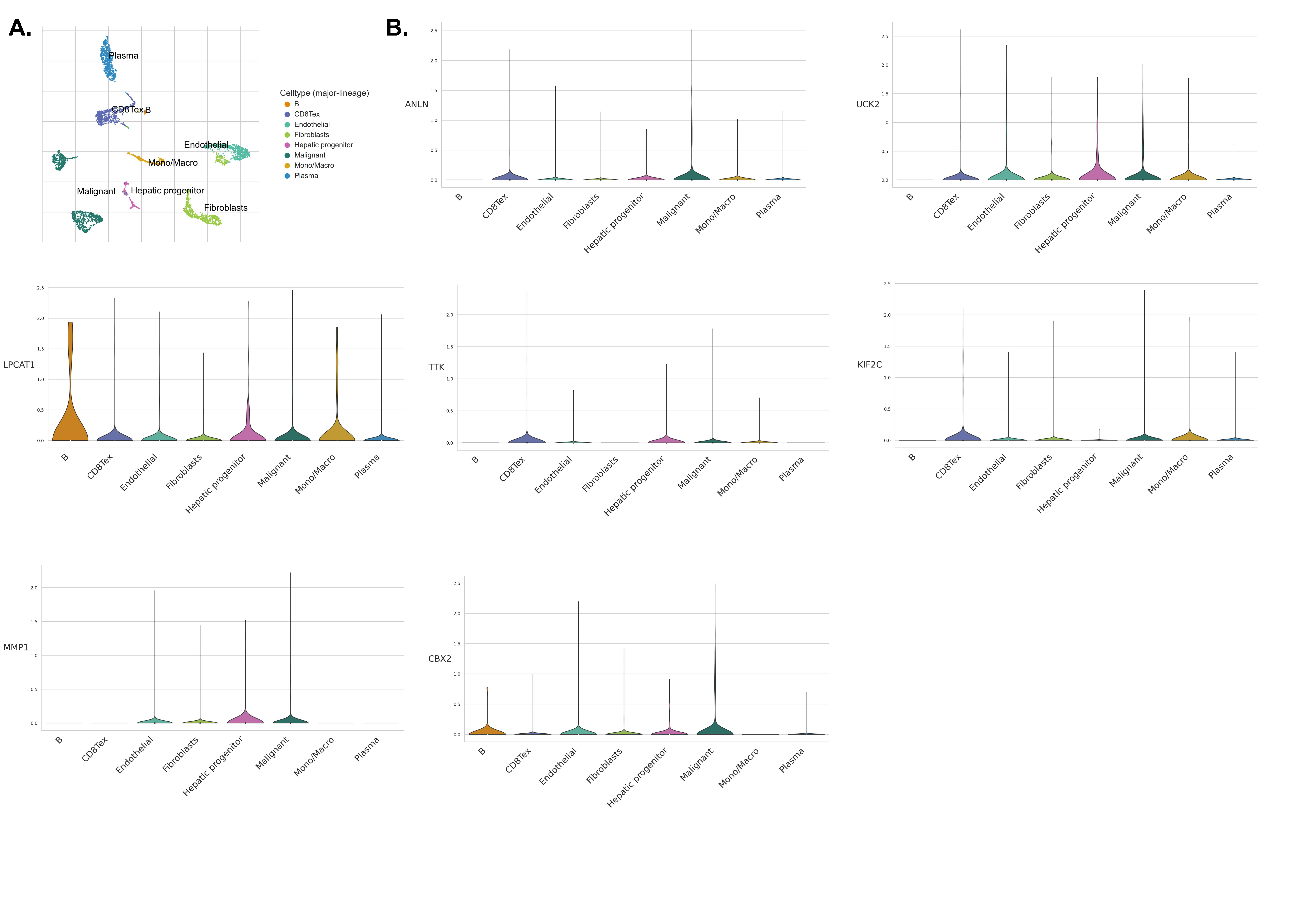

Supplement: Supplementary Figure 5 — Single cell analysis of different genes expression. [file Image_5.tiff]

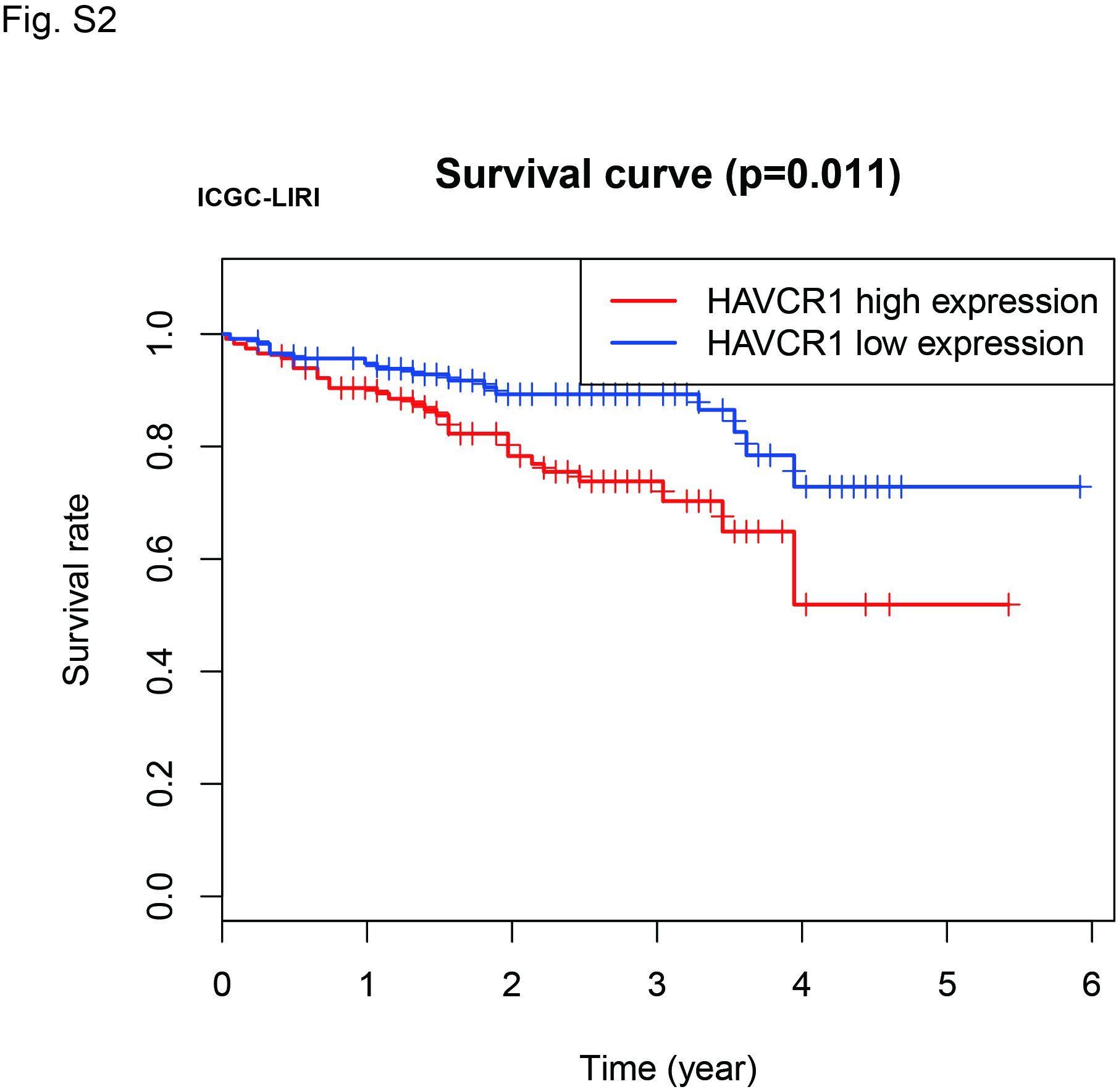

Supplement: Supplementary Figure 6 — KM analysis comparing the high and low expression of HAVCR1 in HCC in the ICGC-LIRI cohort. [file Image_6.jpeg]

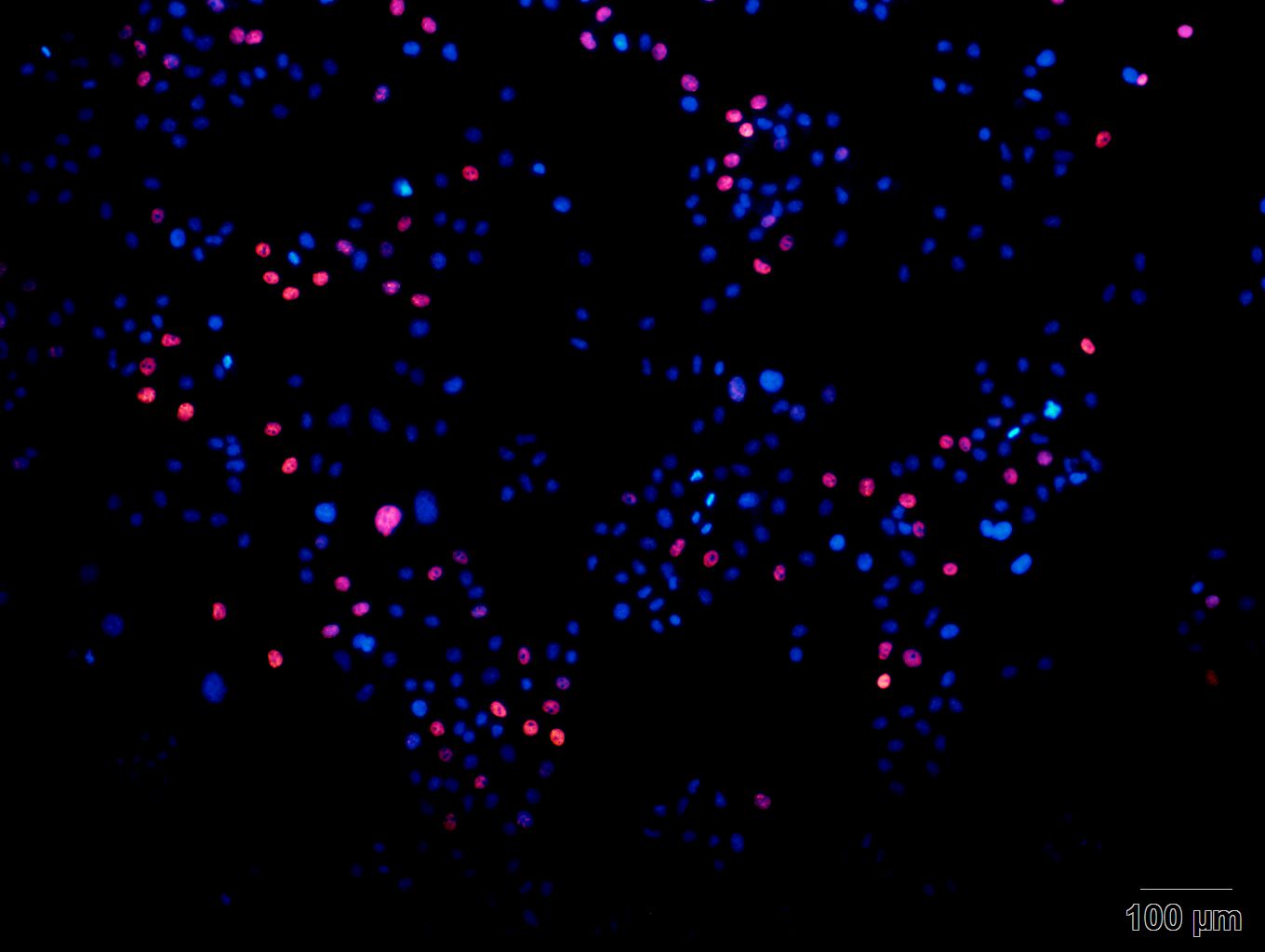

Supplement: Supplementary file 8 [file DataSheet_2.zip › raw data in vitro assay for edior checking/EdU/G2-si1.jpg]

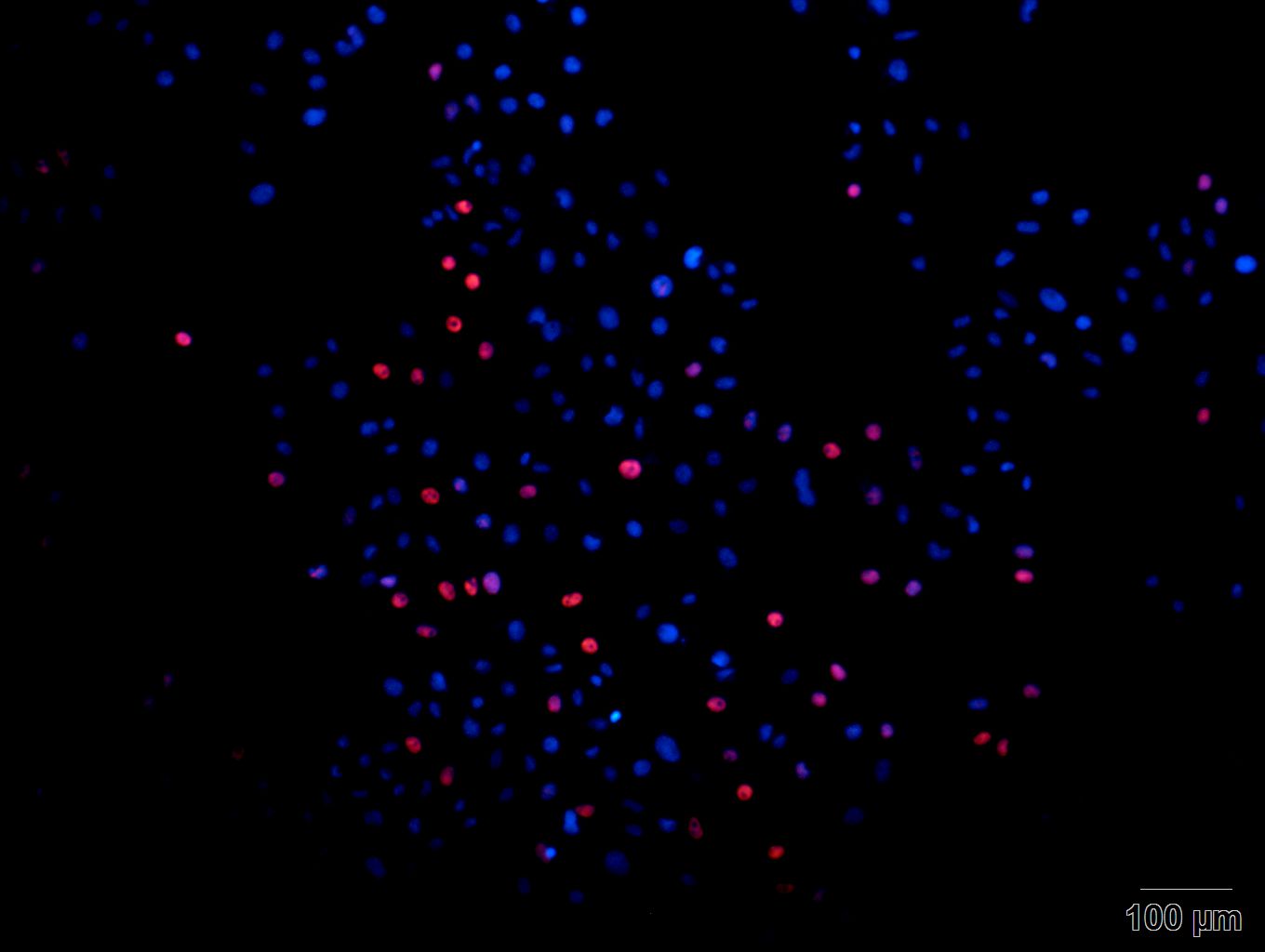

Supplement: Supplementary file 8 [file DataSheet_2.zip › raw data in vitro assay for edior checking/EdU/G2-si2.jpg]

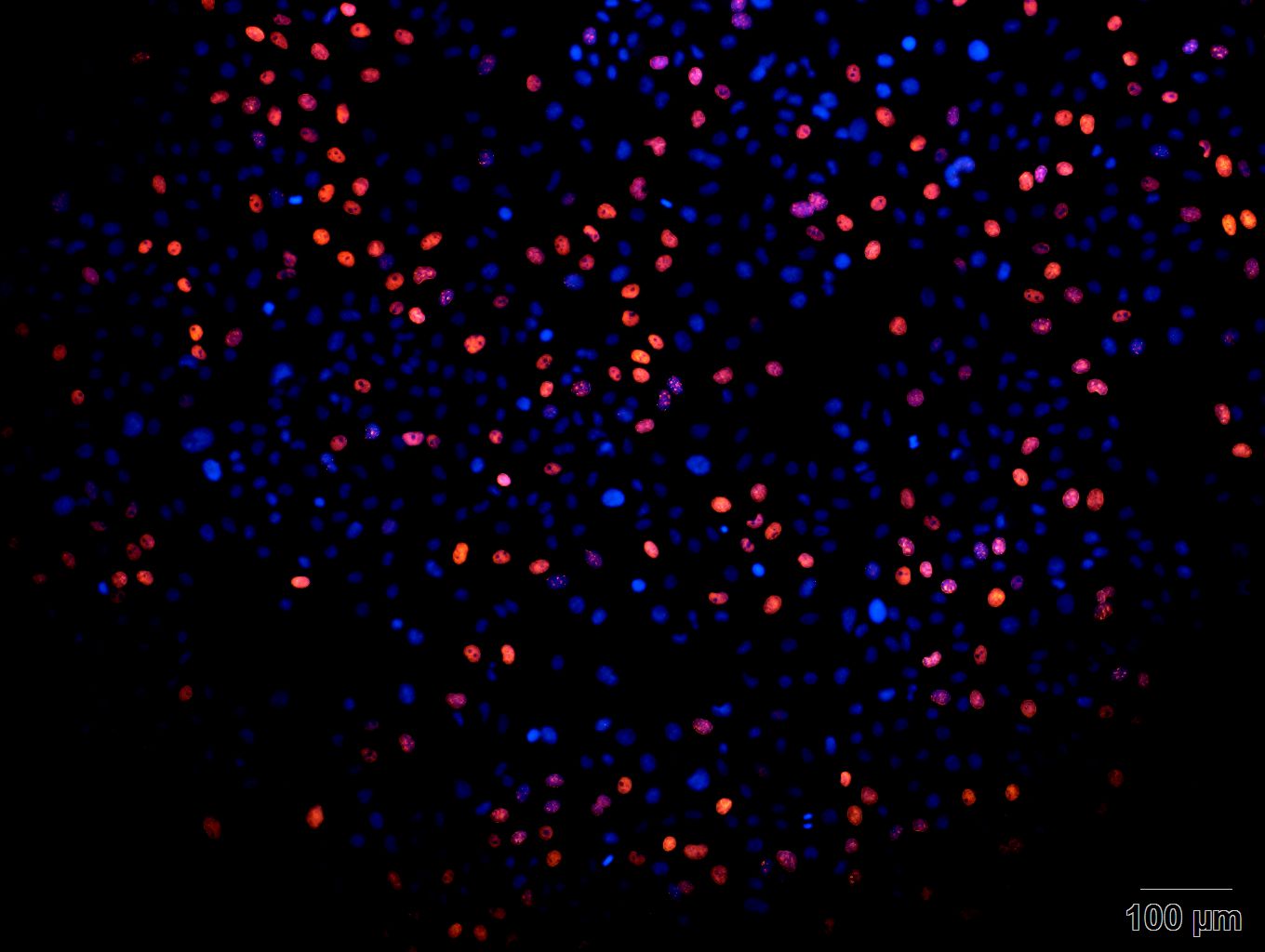

Supplement: Supplementary file 8 [file DataSheet_2.zip › raw data in vitro assay for edior checking/EdU/G2-sicon.jpg]

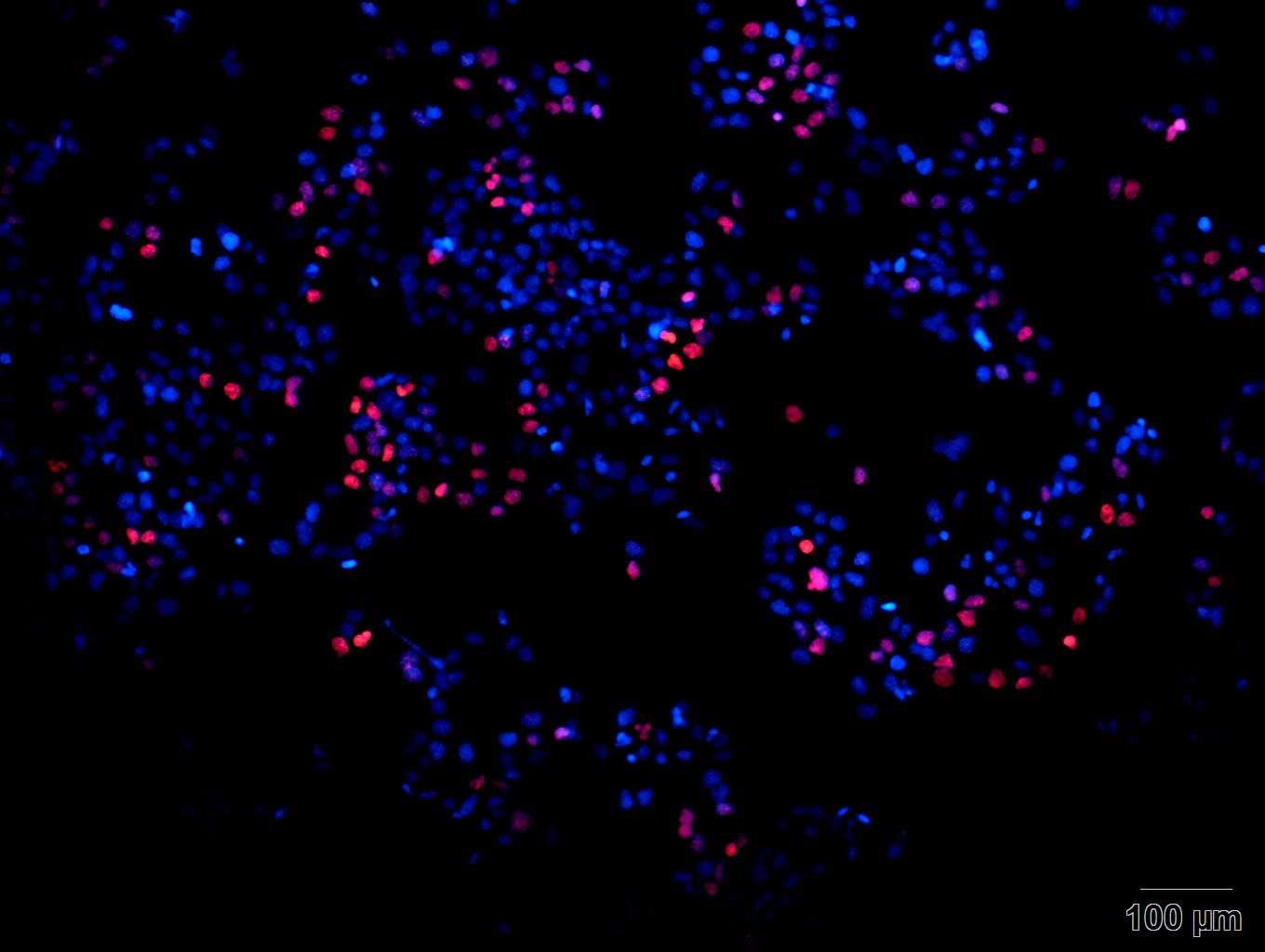

Supplement: Supplementary file 8 [file DataSheet_2.zip › raw data in vitro assay for edior checking/EdU/H7-si1.jpg]

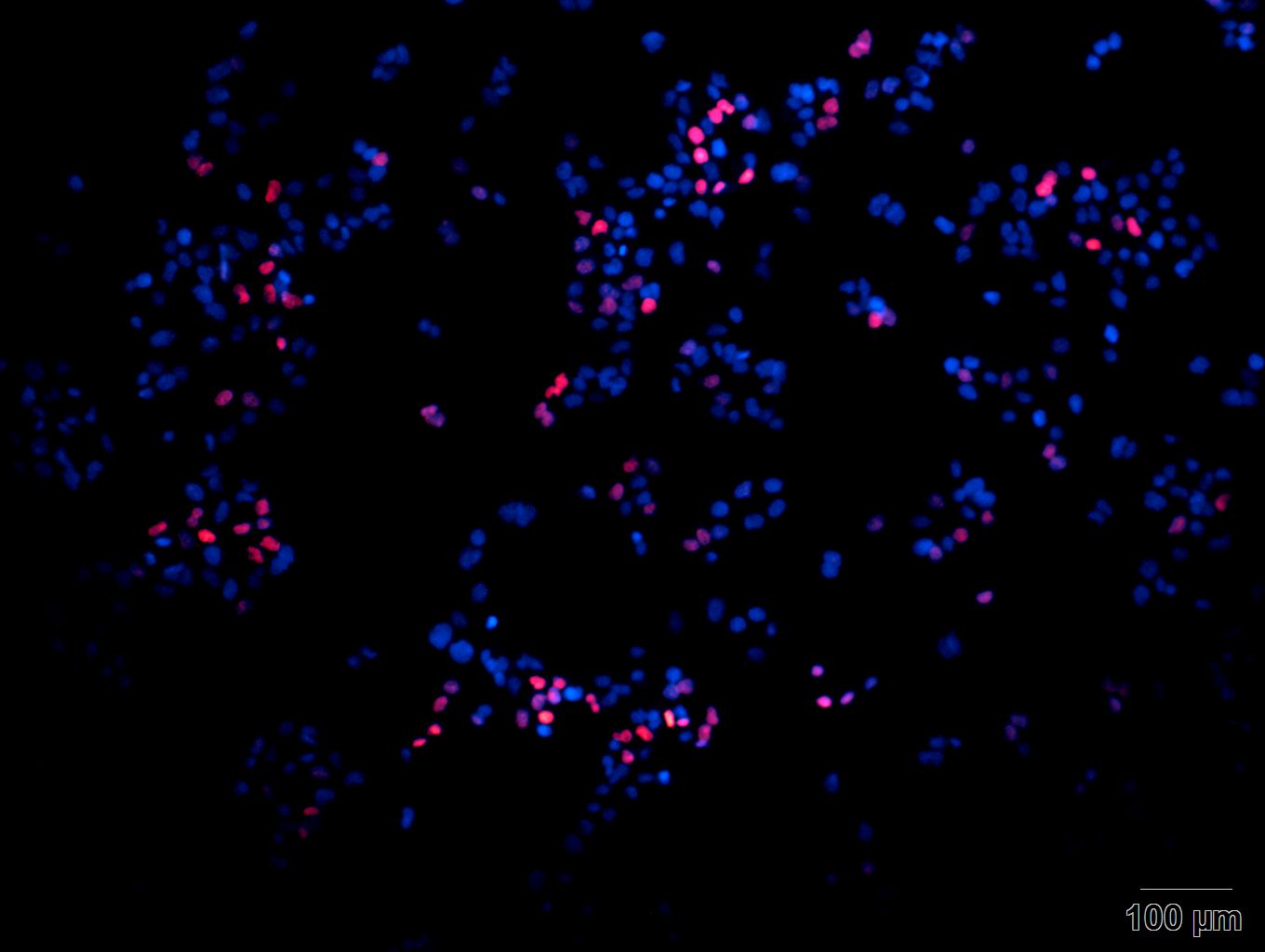

Supplement: Supplementary file 8 [file DataSheet_2.zip › raw data in vitro assay for edior checking/EdU/H7-si2.jpg]

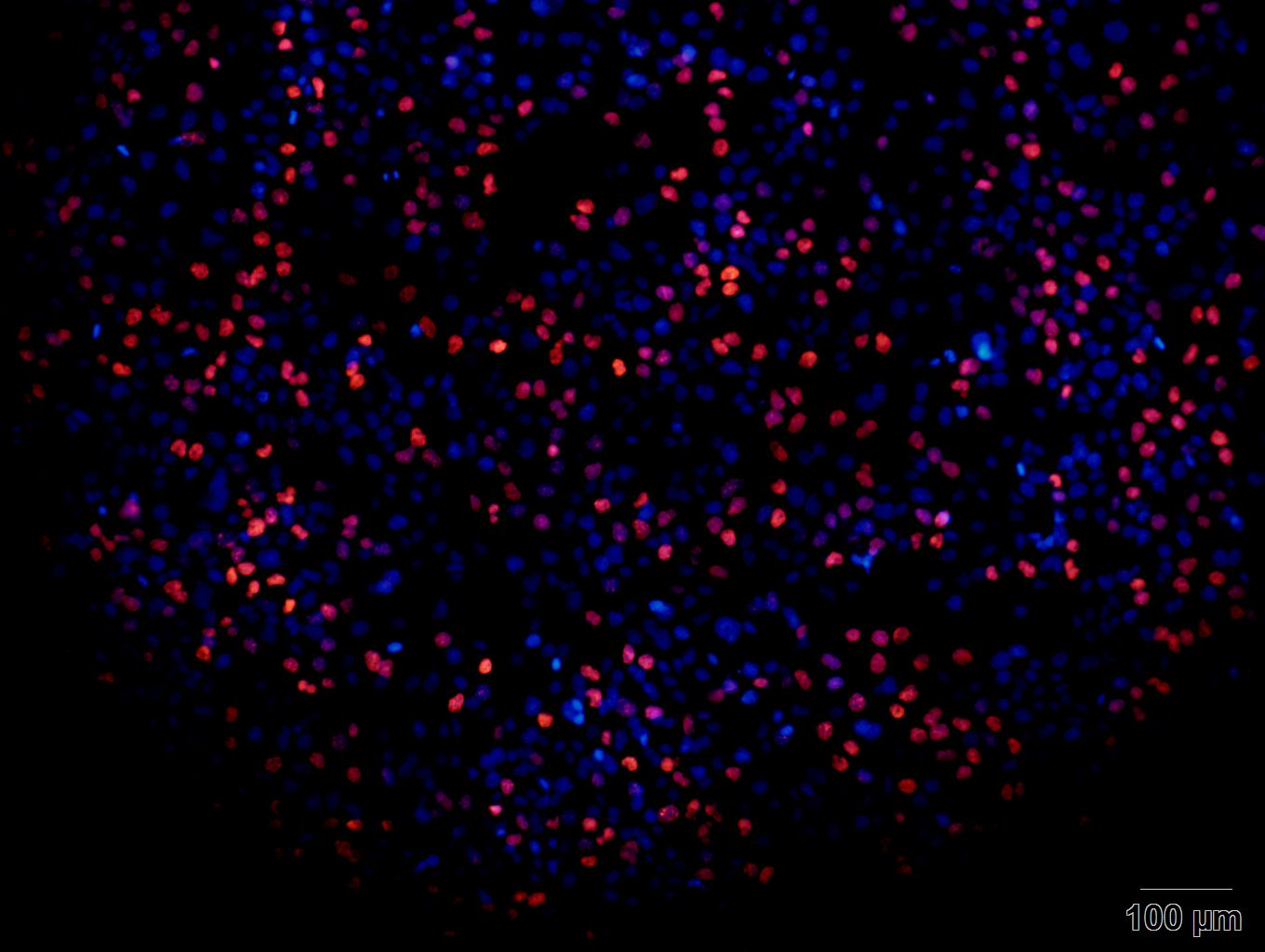

Supplement: Supplementary file 8 [file DataSheet_2.zip › raw data in vitro assay for edior checking/EdU/H7-sicon.jpg]

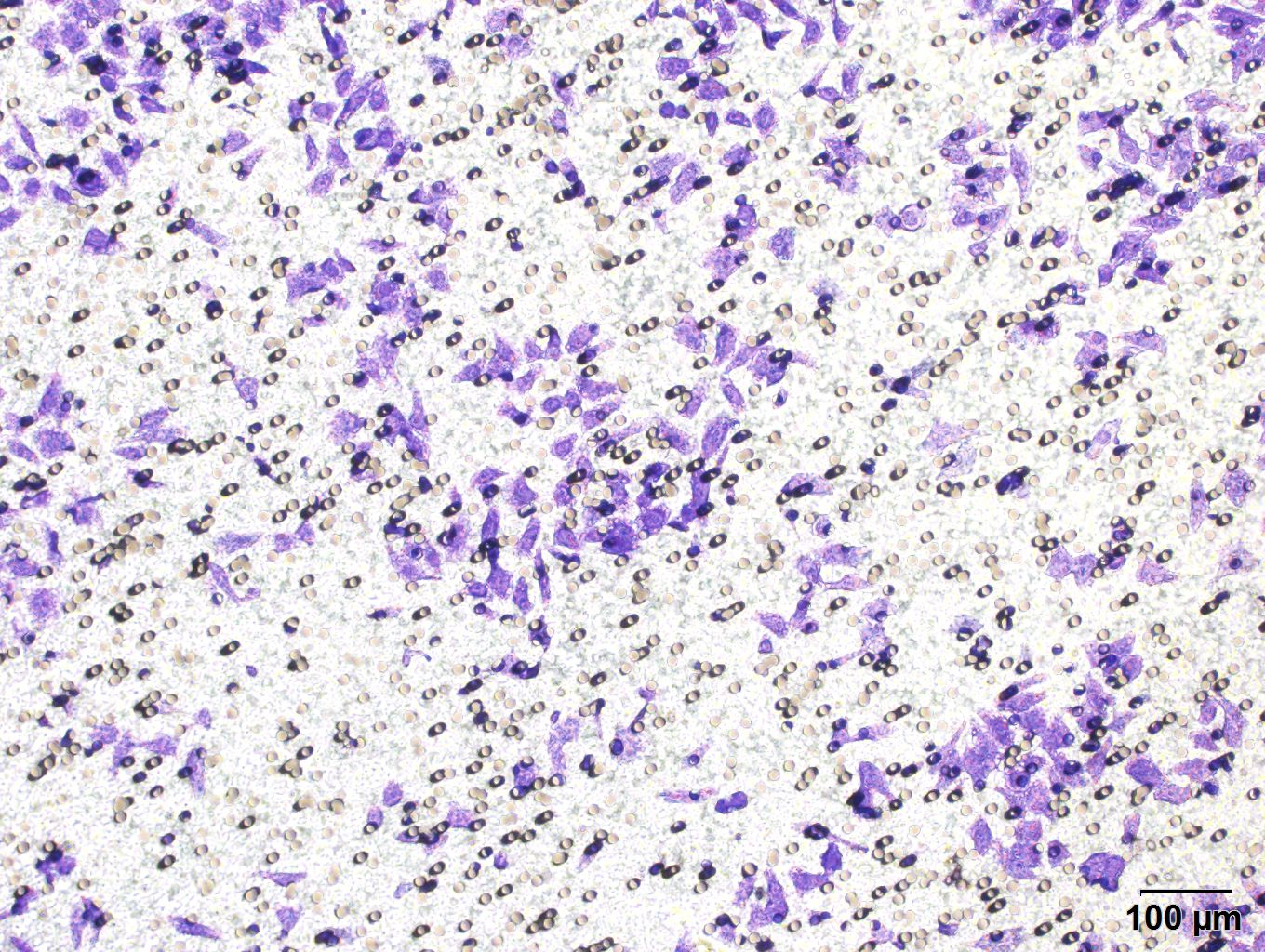

Supplement: Supplementary file 8 [file DataSheet_2.zip › raw data in vitro assay for edior checking/Matrigel Transwell/HepG2/si1.jpg]

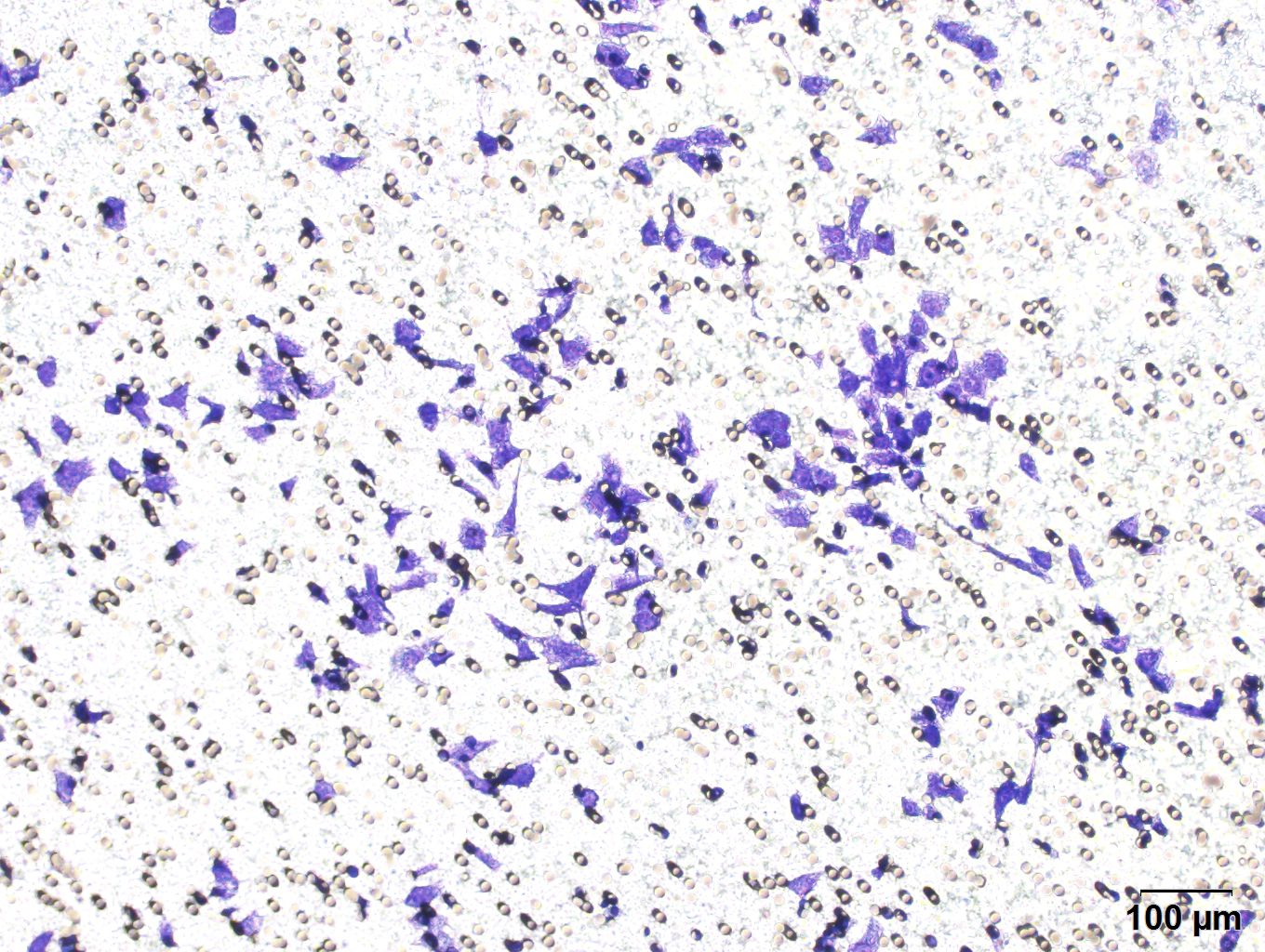

Supplement: Supplementary file 8 [file DataSheet_2.zip › raw data in vitro assay for edior checking/Matrigel Transwell/HepG2/si2.jpg]

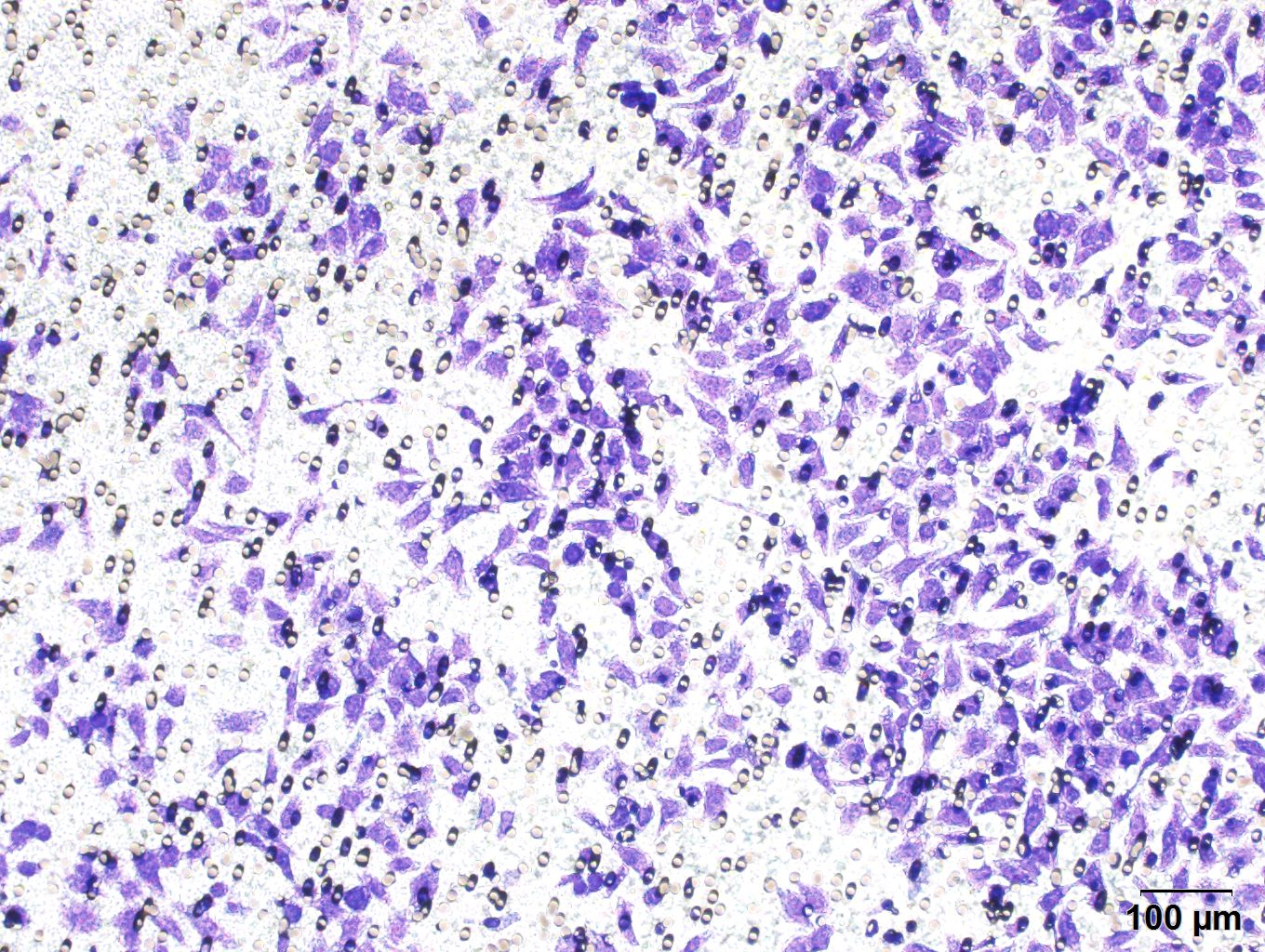

Supplement: Supplementary file 8 [file DataSheet_2.zip › raw data in vitro assay for edior checking/Matrigel Transwell/HepG2/sicon.jpg]

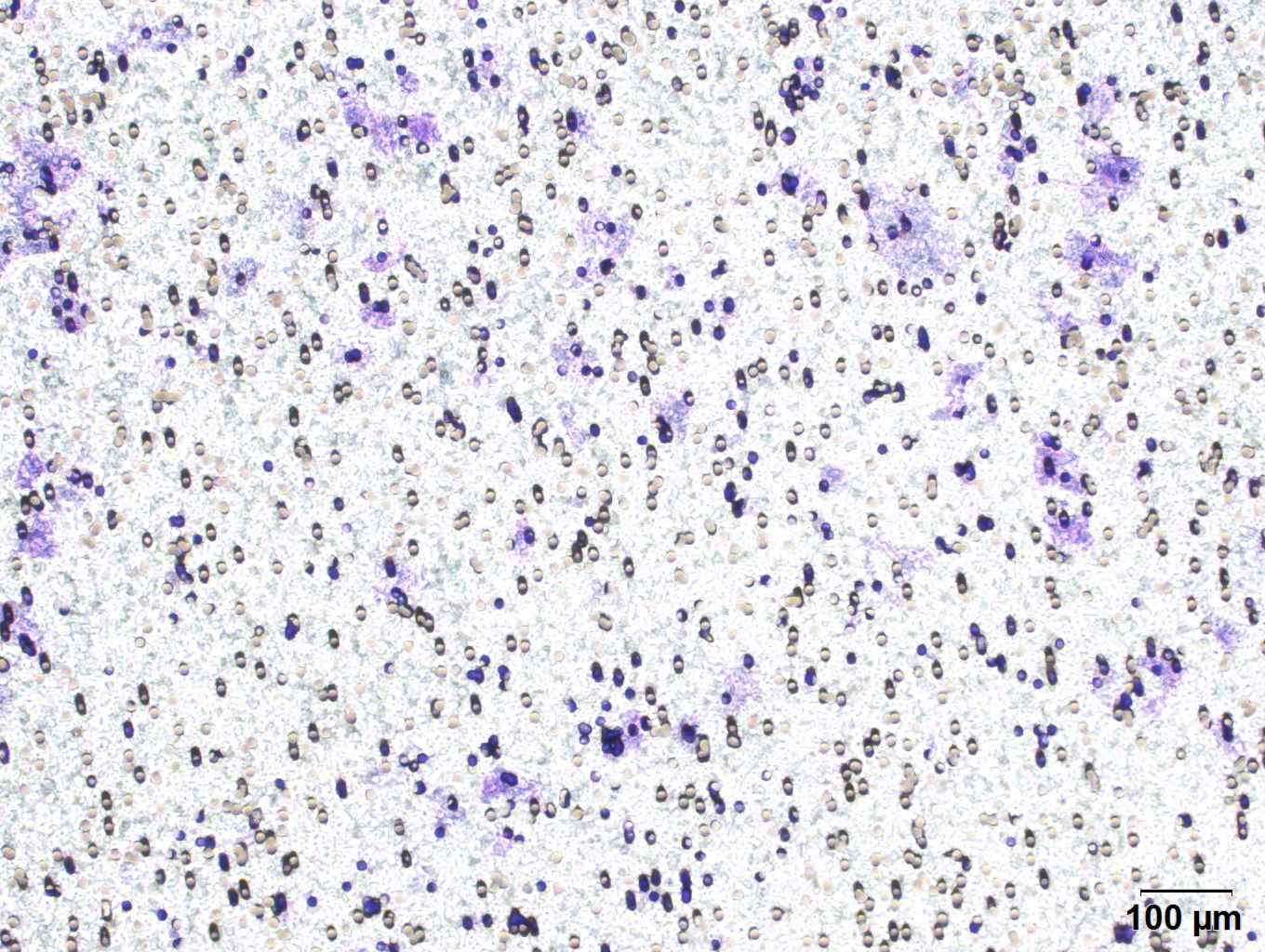

Supplement: Supplementary file 8 [file DataSheet_2.zip › raw data in vitro assay for edior checking/Matrigel Transwell/Huh7/si1.jpg]

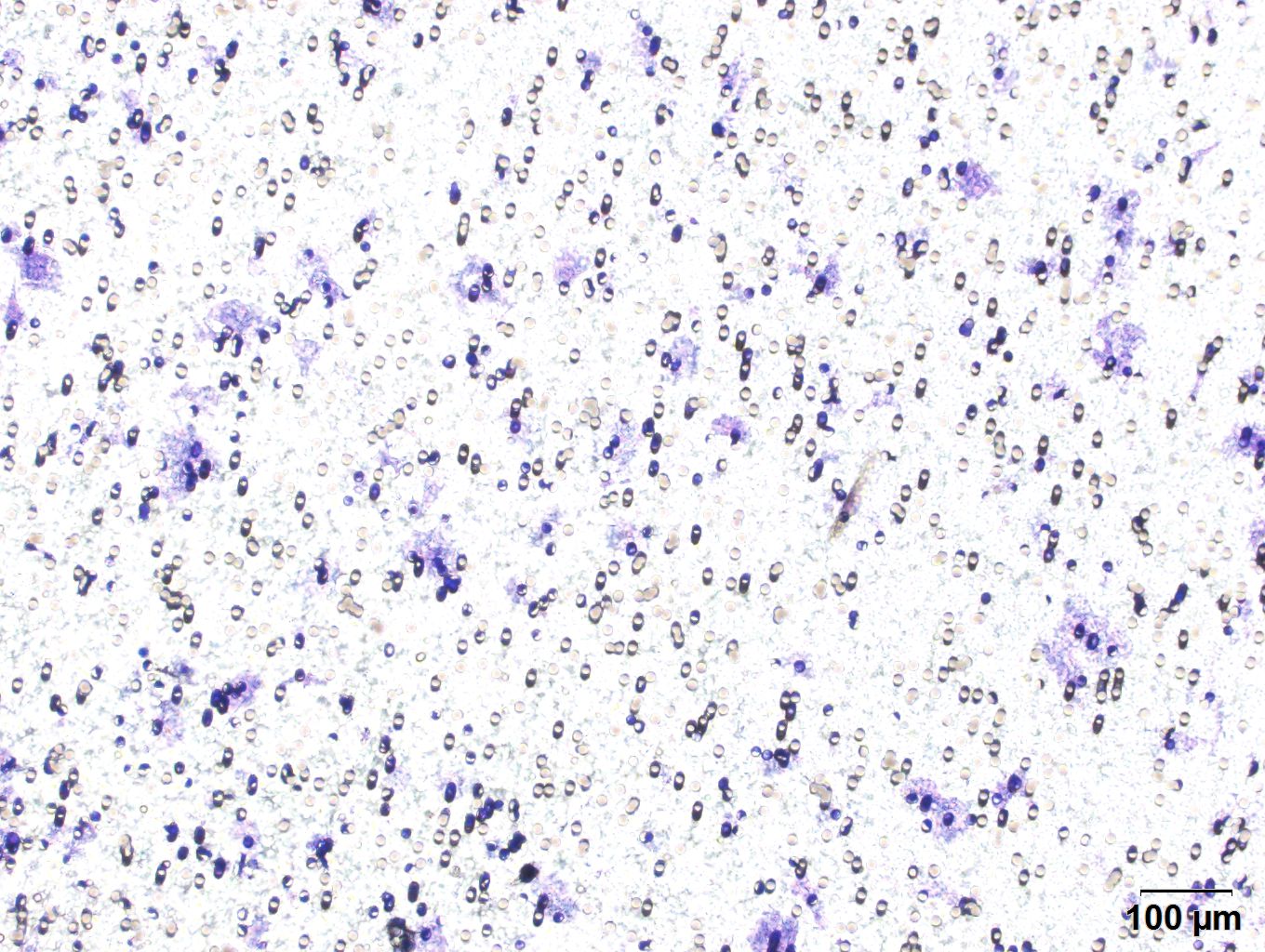

Supplement: Supplementary file 8 [file DataSheet_2.zip › raw data in vitro assay for edior checking/Matrigel Transwell/Huh7/si2.jpg]

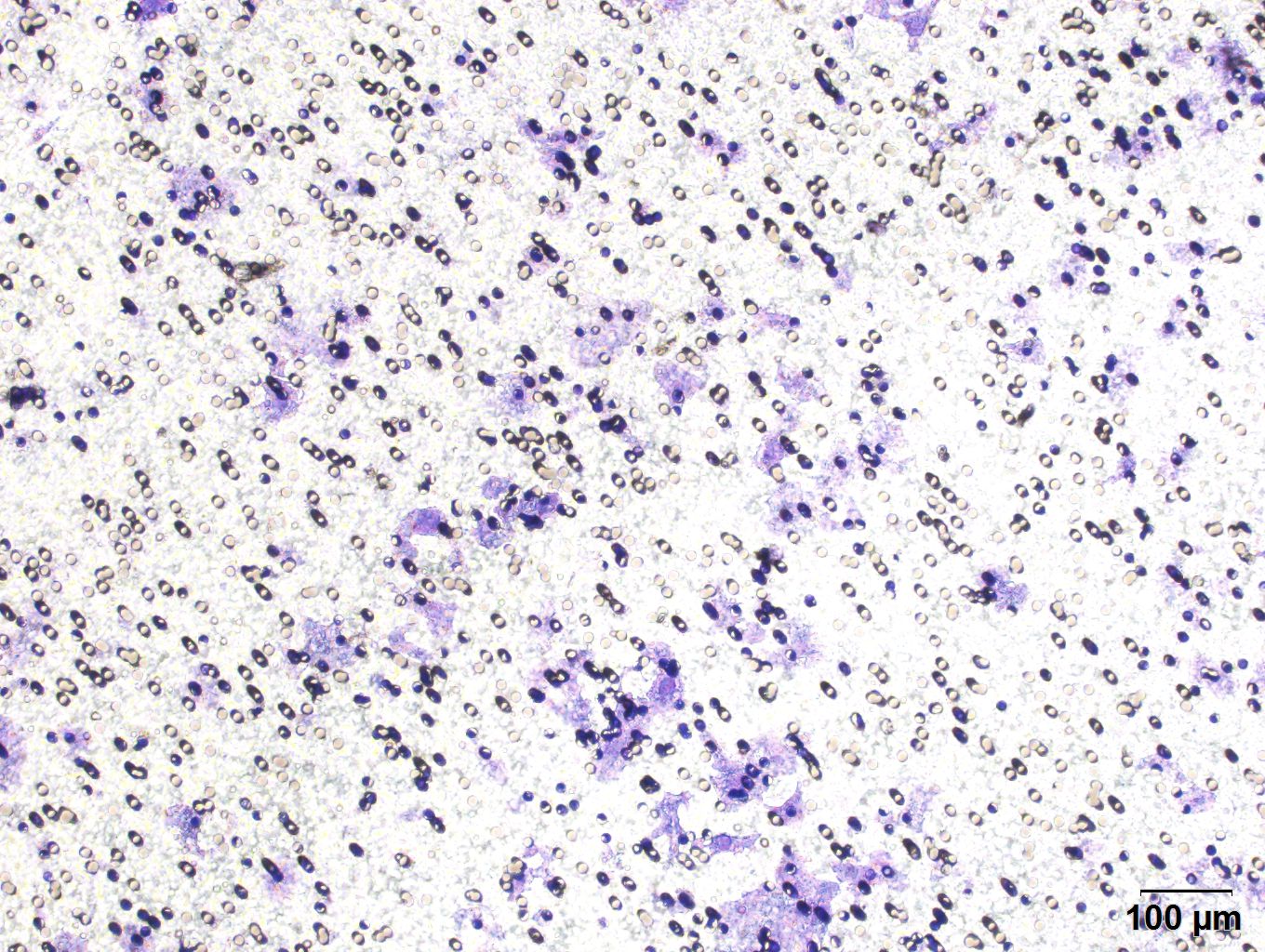

Supplement: Supplementary file 8 [file DataSheet_2.zip › raw data in vitro assay for edior checking/Matrigel Transwell/Huh7/sicon.jpg]

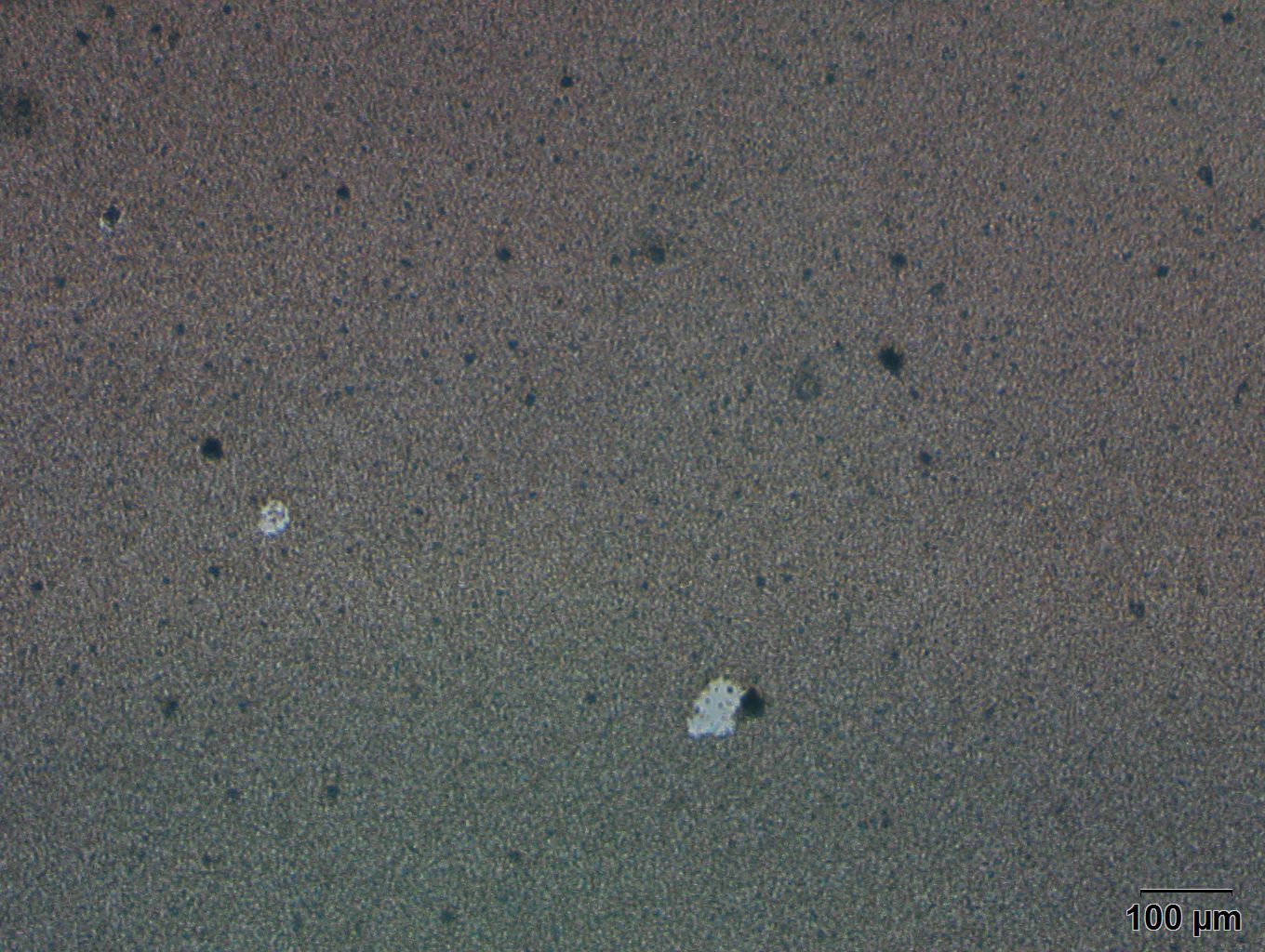

Supplement: Supplementary file 8 [file DataSheet_2.zip › raw data in vitro assay for edior checking/mobility/si1-G2.jpg]

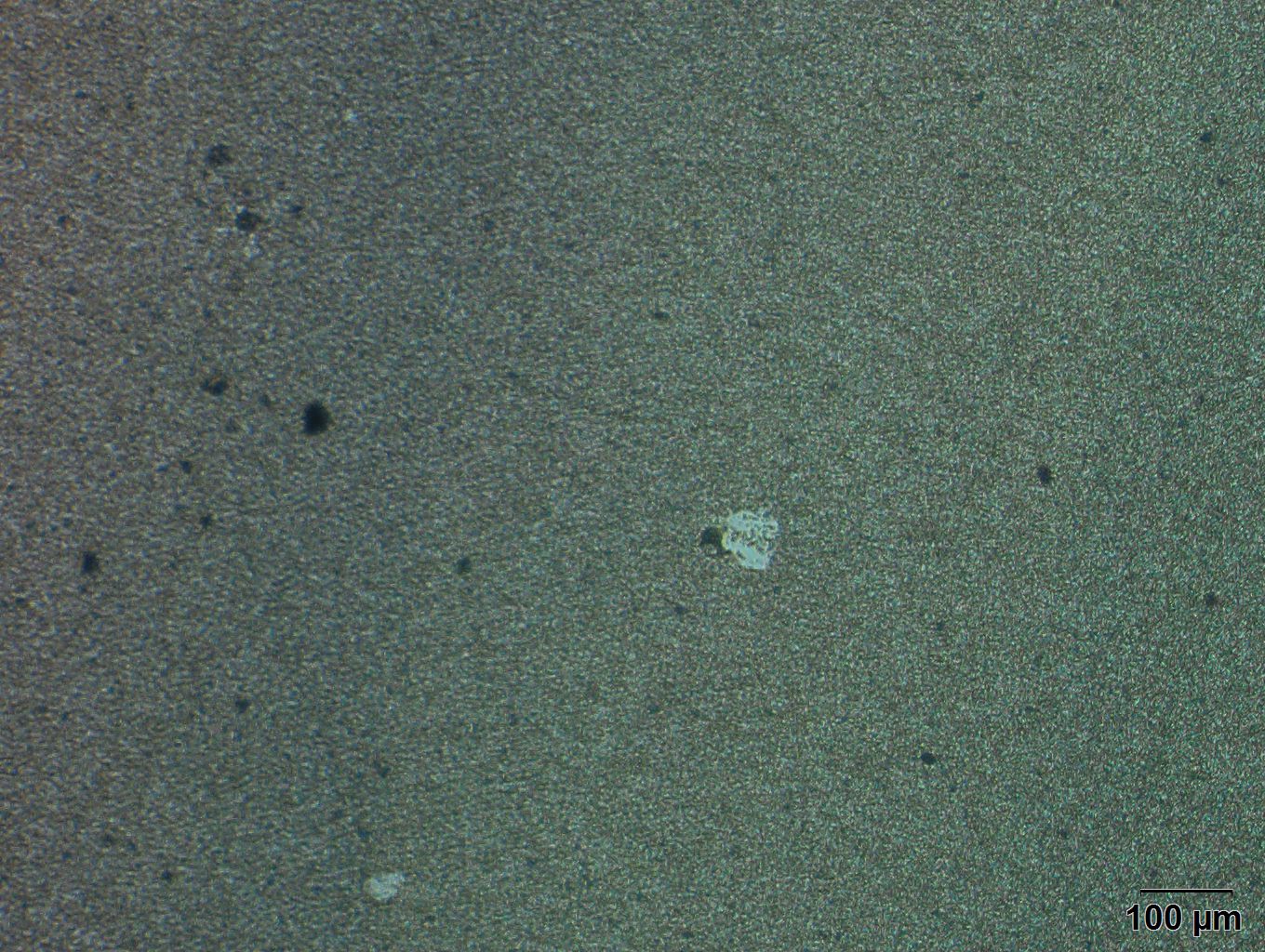

Supplement: Supplementary file 8 [file DataSheet_2.zip › raw data in vitro assay for edior checking/mobility/si1-H7.jpg]

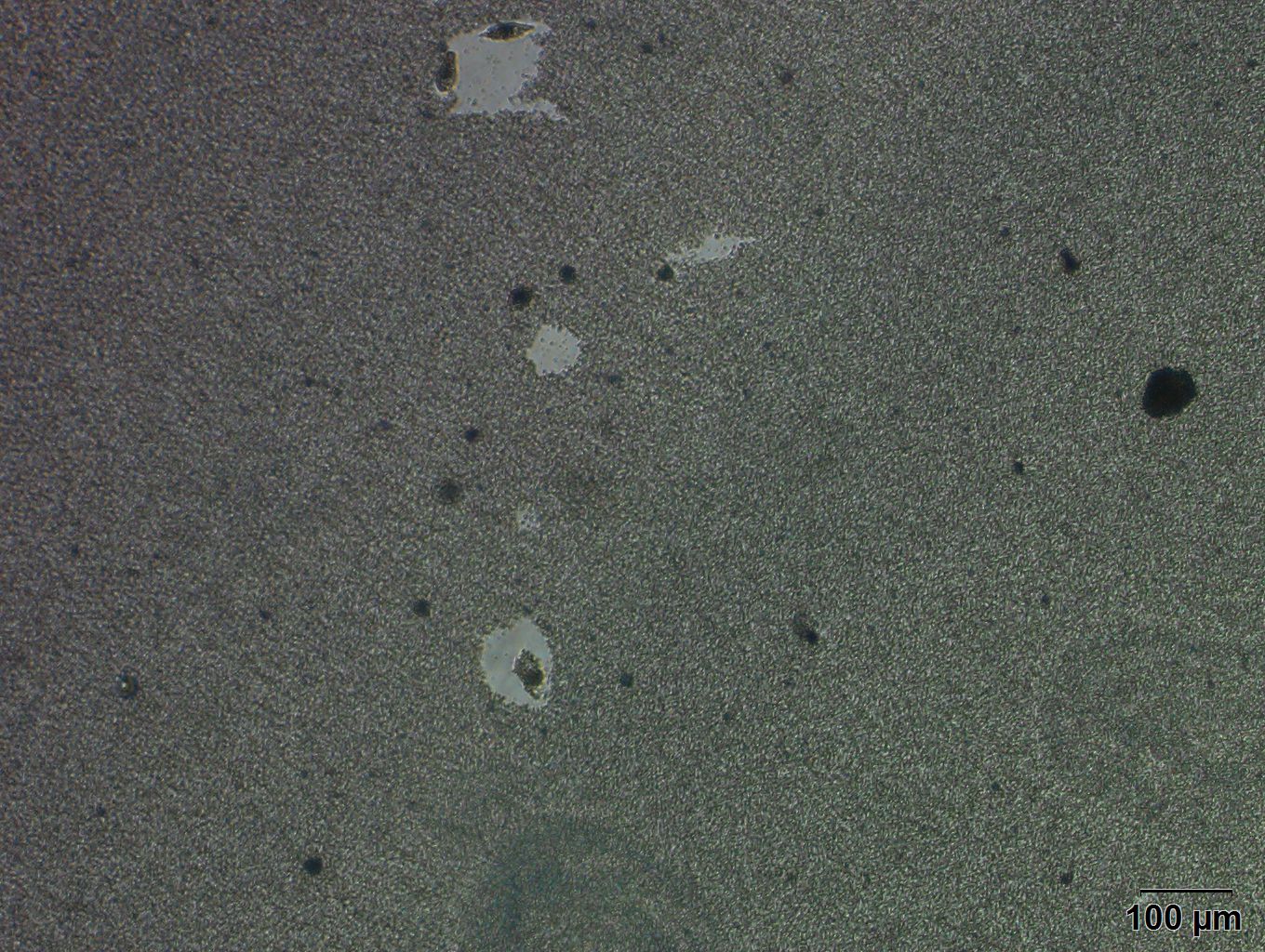

Supplement: Supplementary file 8 [file DataSheet_2.zip › raw data in vitro assay for edior checking/mobility/si2-G2.jpg]

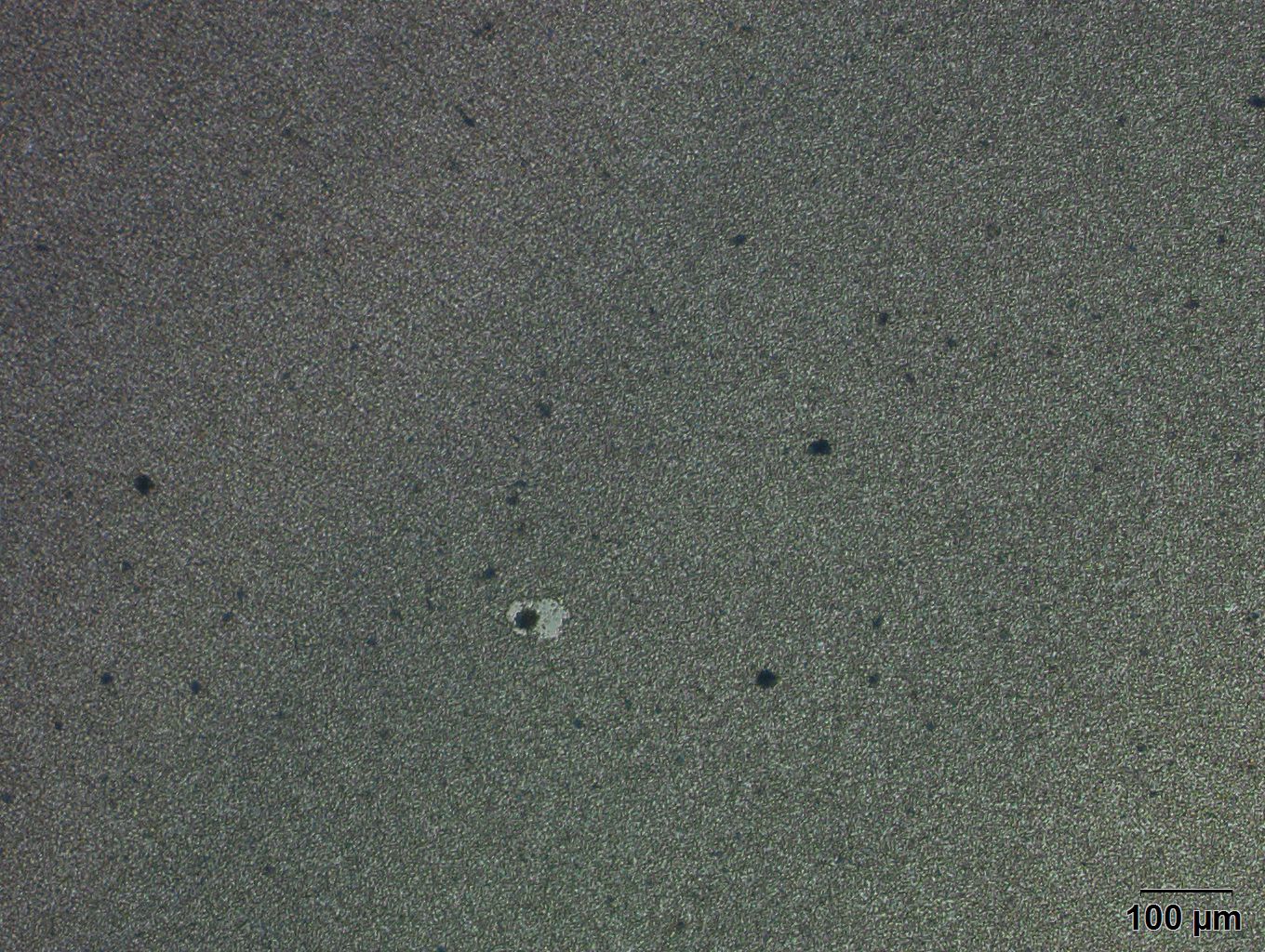

Supplement: Supplementary file 8 [file DataSheet_2.zip › raw data in vitro assay for edior checking/mobility/si2-H7.jpg]

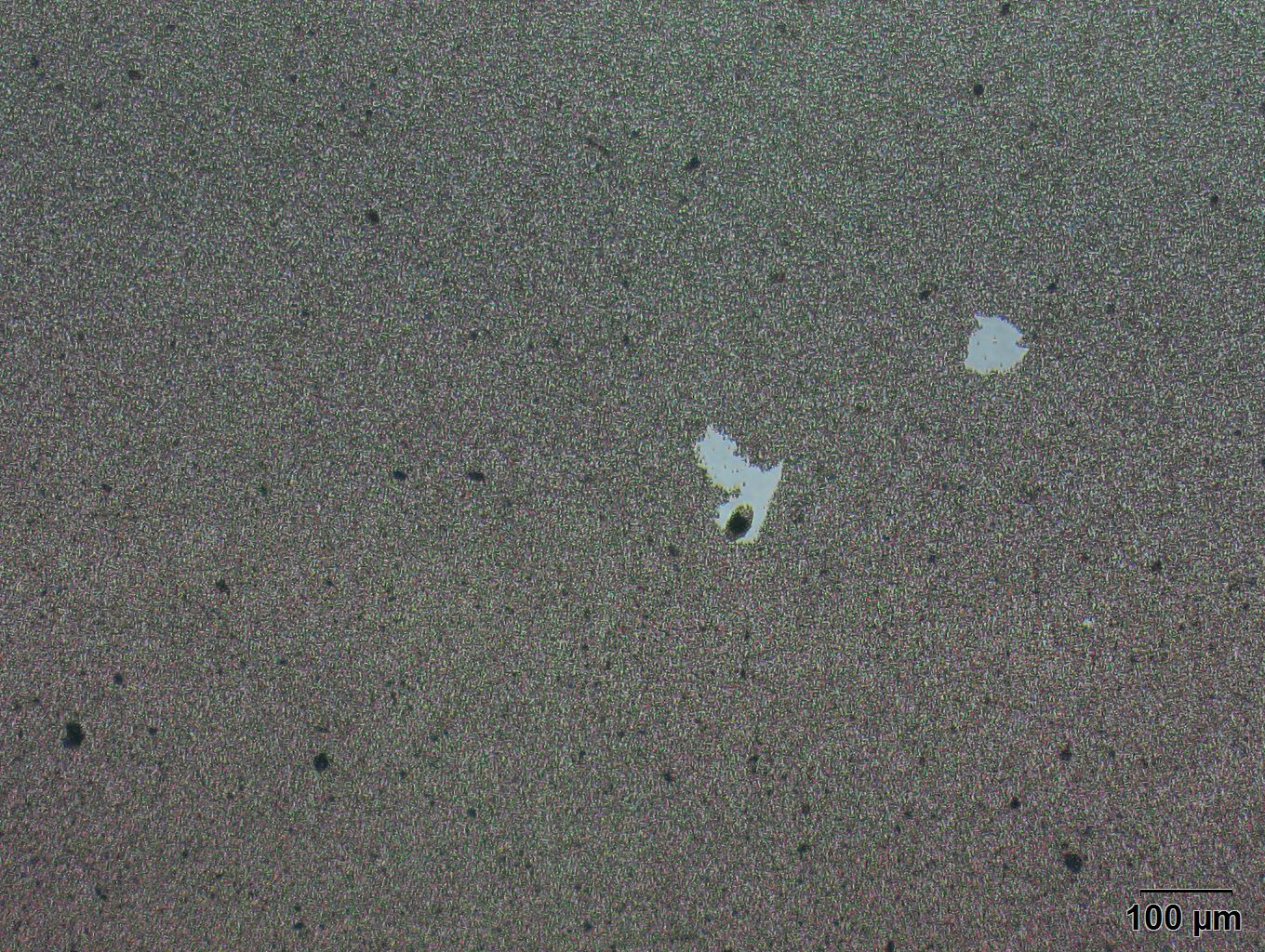

Supplement: Supplementary file 8 [file DataSheet_2.zip › raw data in vitro assay for edior checking/mobility/sicon-G2.jpg]

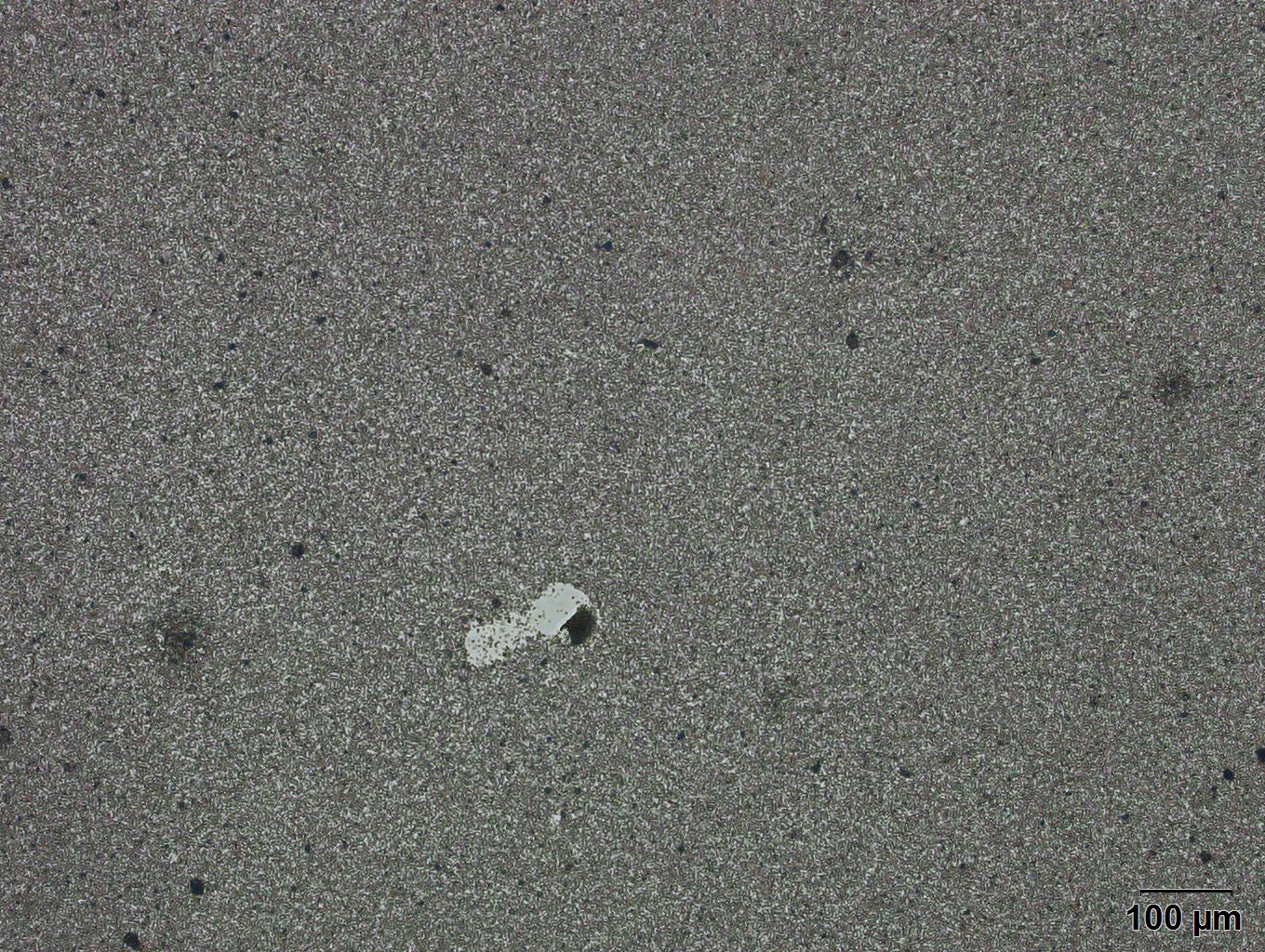

Supplement: Supplementary file 8 [file DataSheet_2.zip › raw data in vitro assay for edior checking/mobility/sicon-H7.jpg]

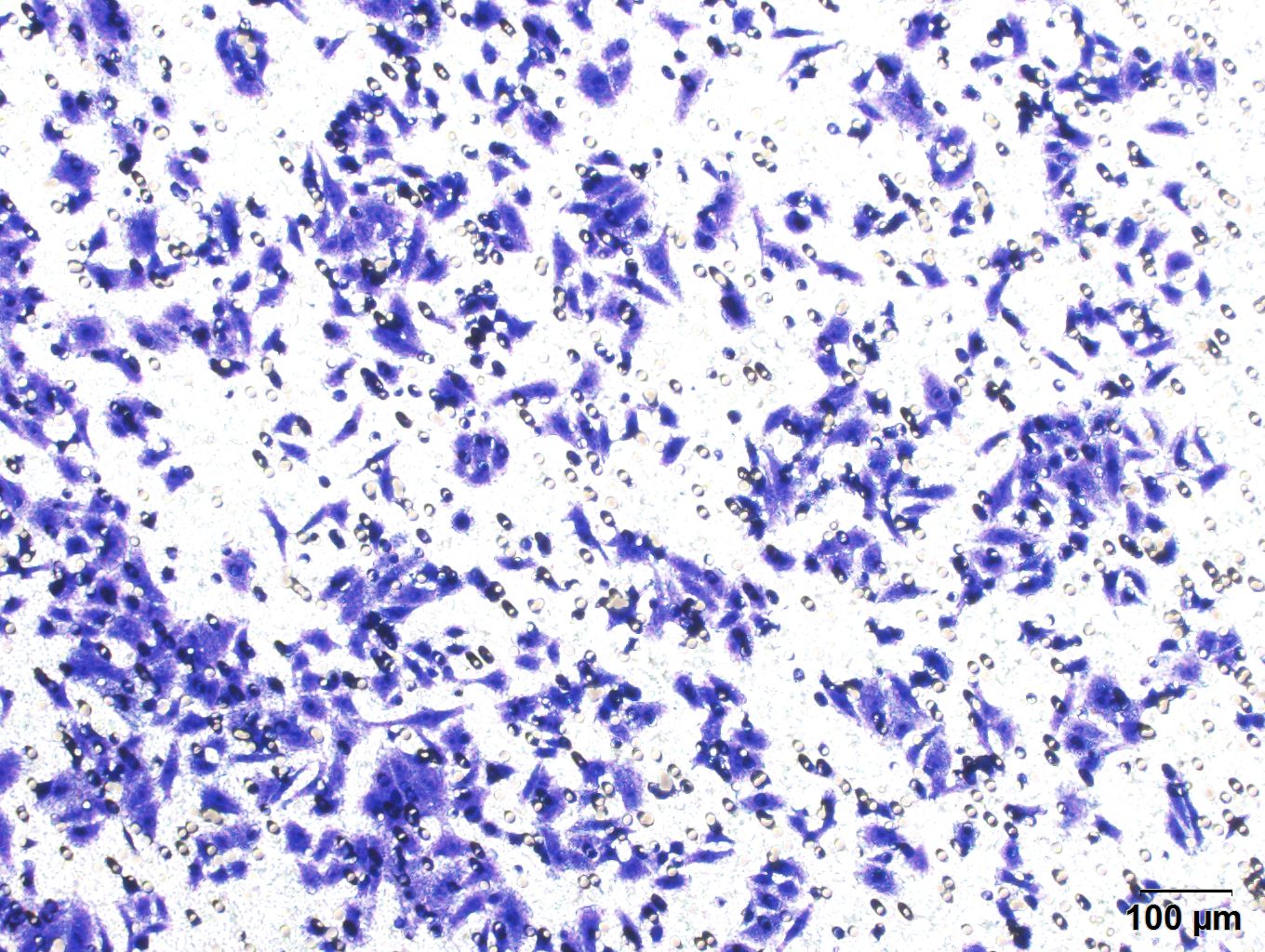

Supplement: Supplementary file 8 [file DataSheet_2.zip › raw data in vitro assay for edior checking/Transwell/G2/si1.jpg]

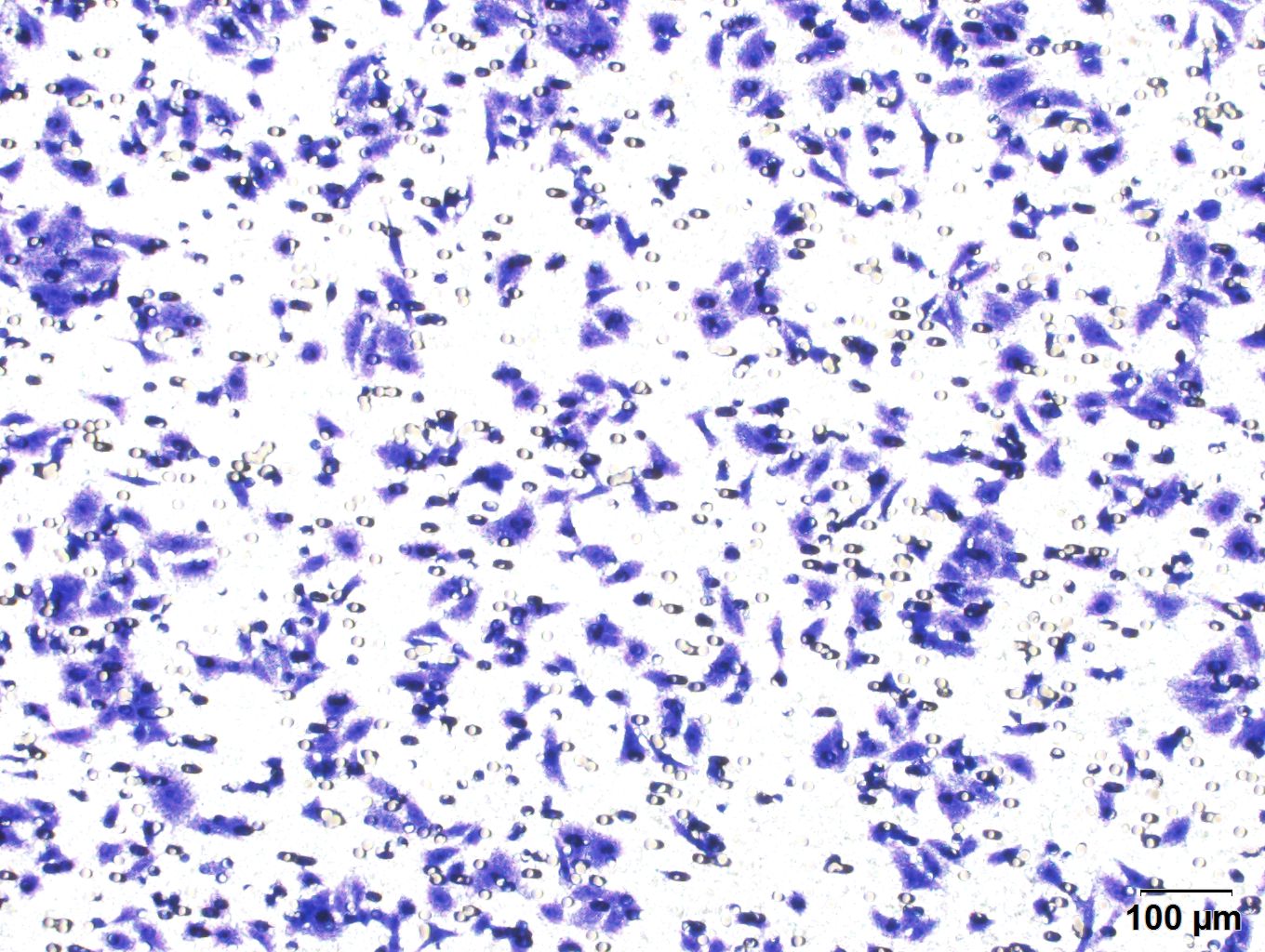

Supplement: Supplementary file 8 [file DataSheet_2.zip › raw data in vitro assay for edior checking/Transwell/G2/si2.jpg]

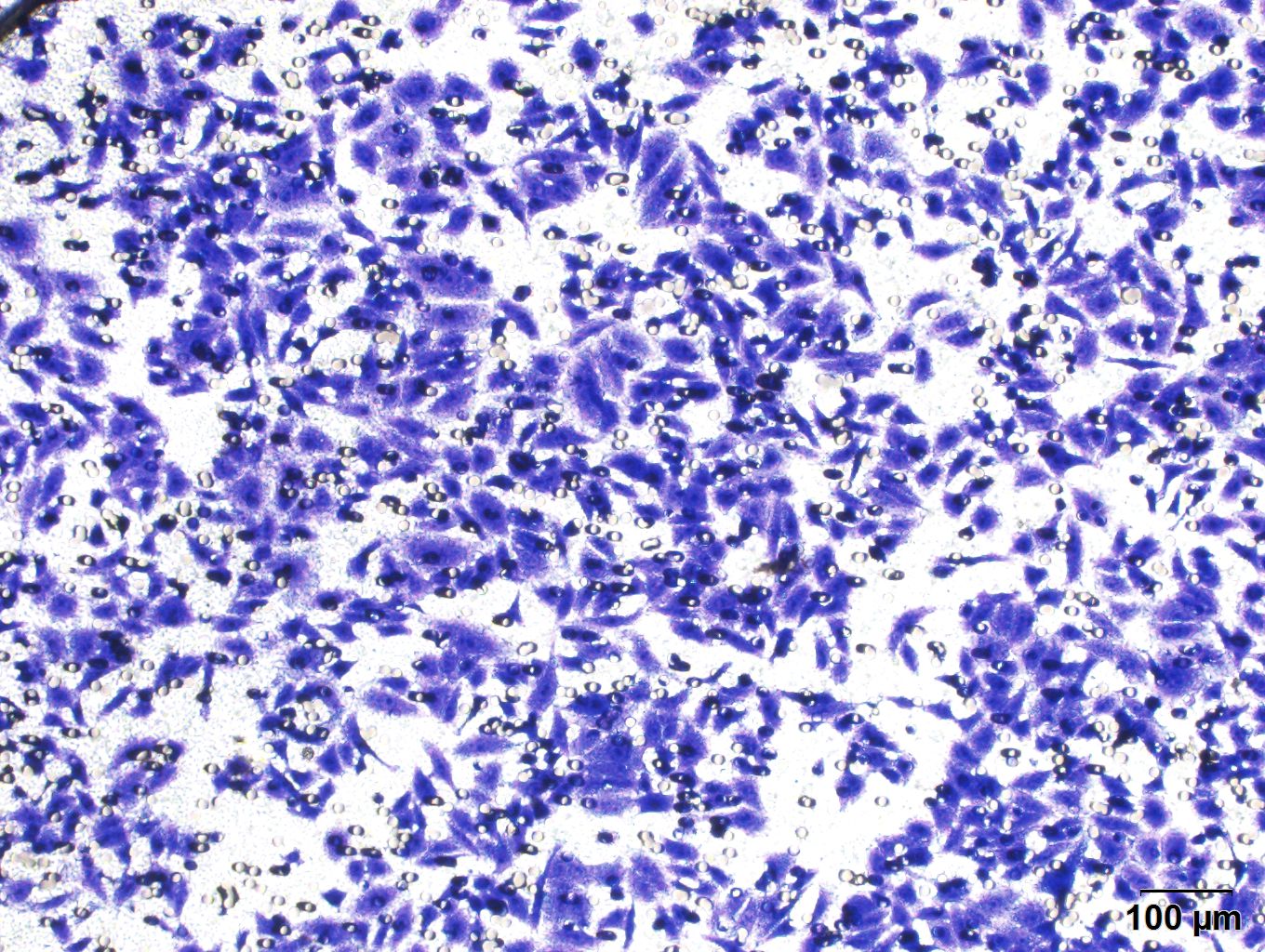

Supplement: Supplementary file 8 [file DataSheet_2.zip › raw data in vitro assay for edior checking/Transwell/G2/sicon.jpg]

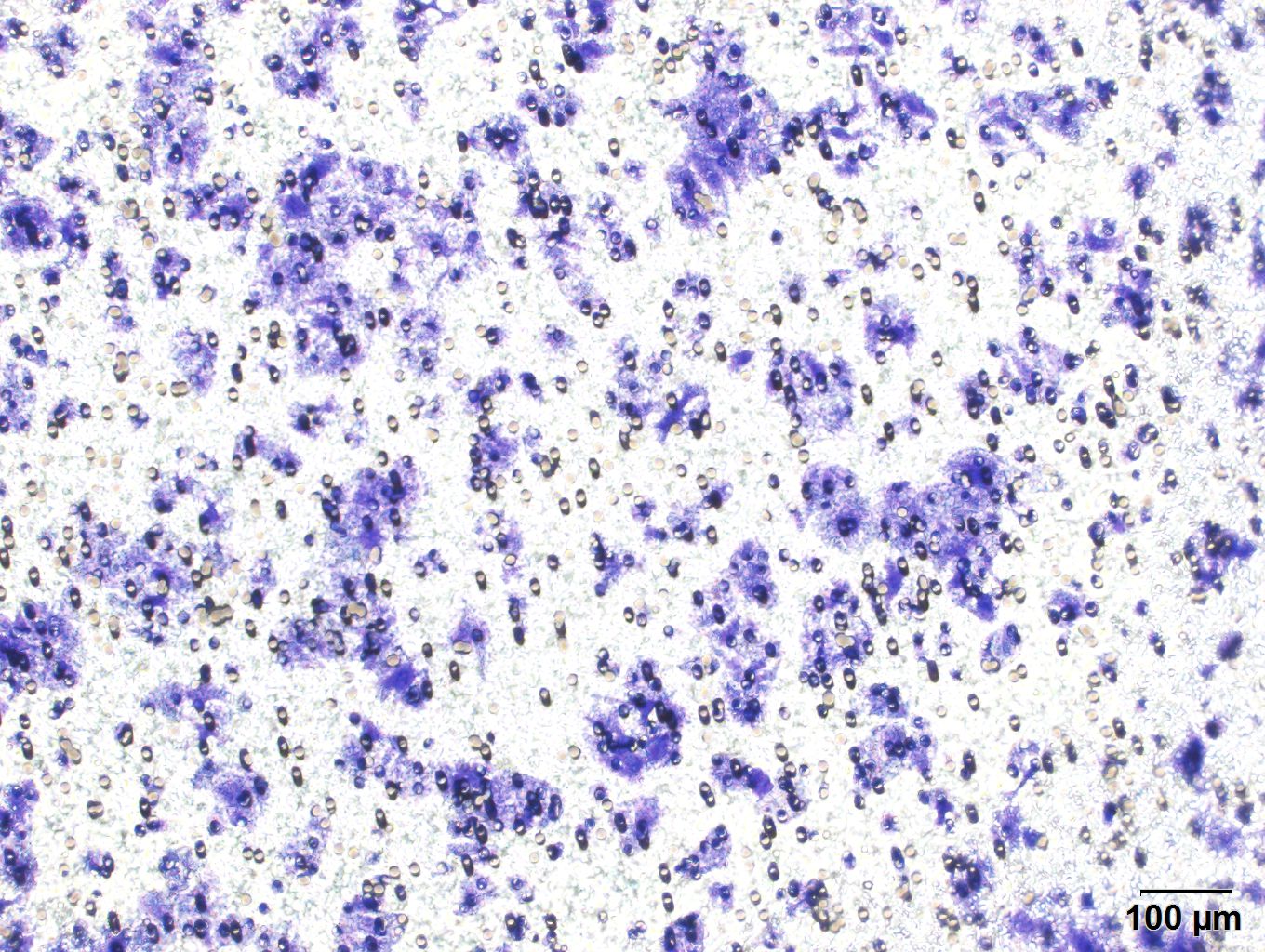

Supplement: Supplementary file 8 [file DataSheet_2.zip › raw data in vitro assay for edior checking/Transwell/Hu7/si1.jpg]

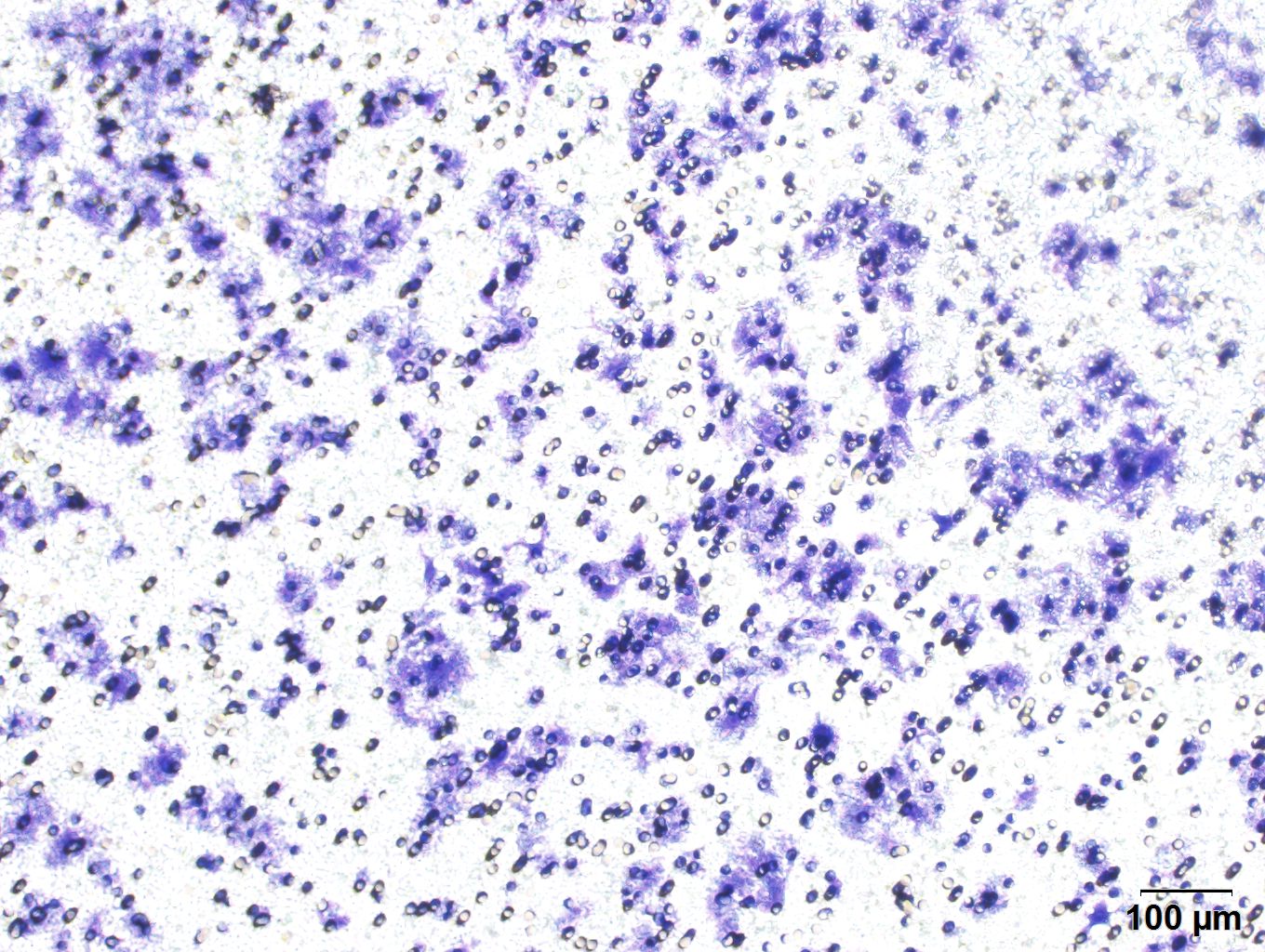

Supplement: Supplementary file 8 [file DataSheet_2.zip › raw data in vitro assay for edior checking/Transwell/Hu7/si2.jpg]

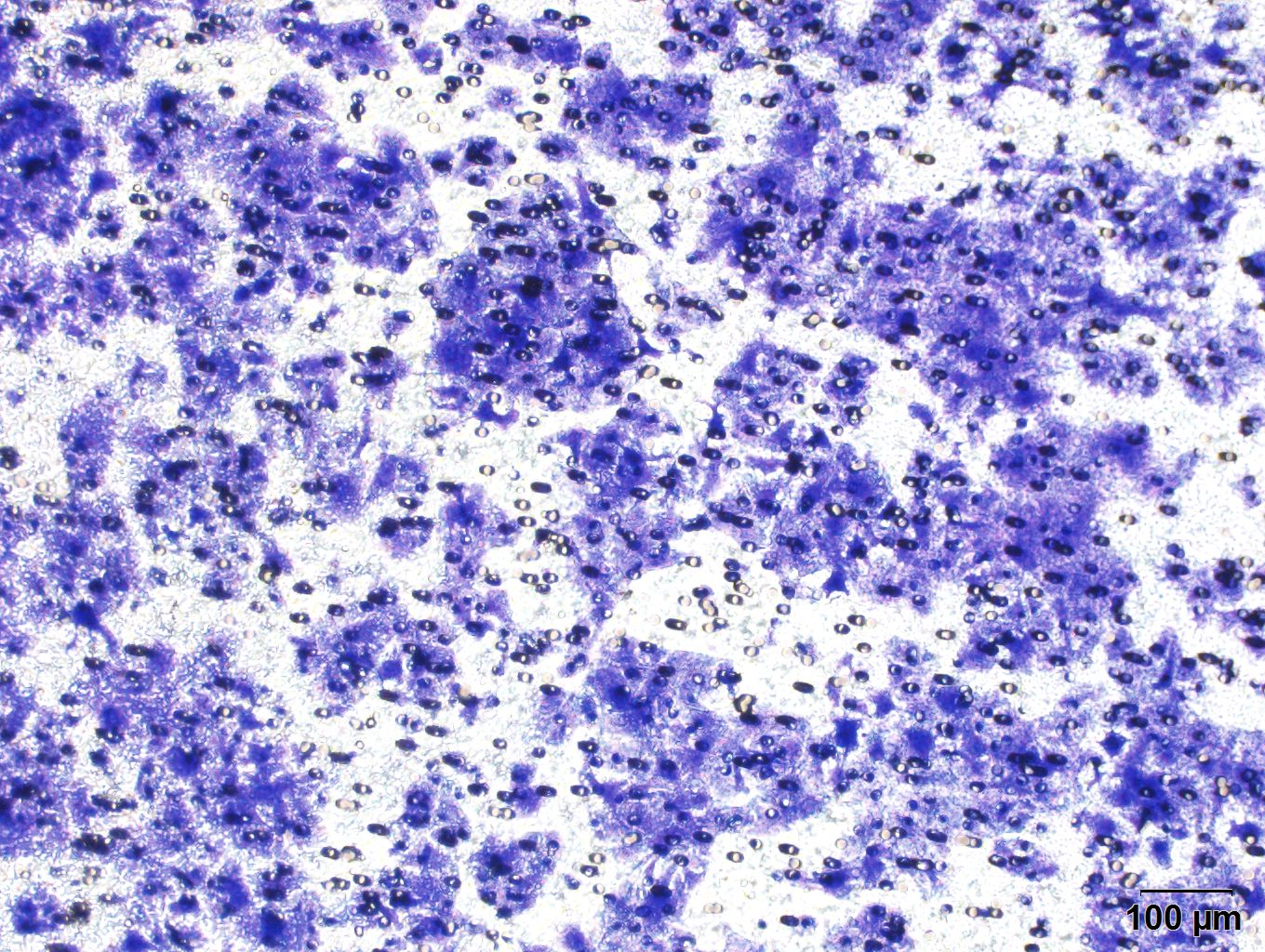

Supplement: Supplementary file 8 [file DataSheet_2.zip › raw data in vitro assay for edior checking/Transwell/Hu7/sicon.jpg]

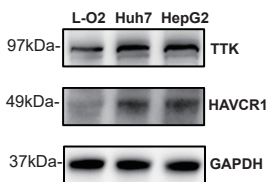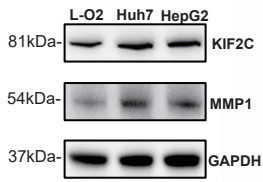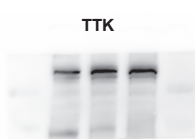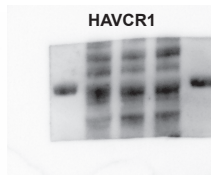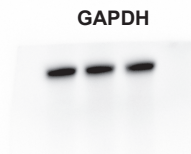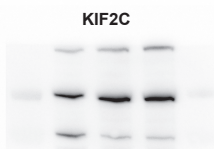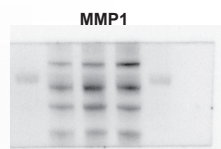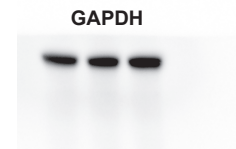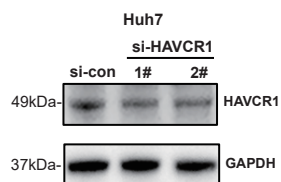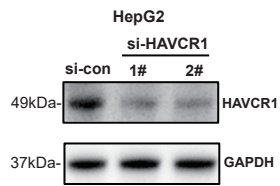

Huh7

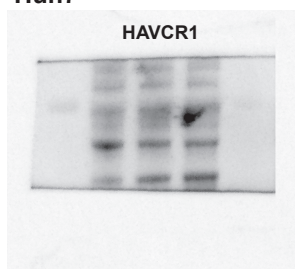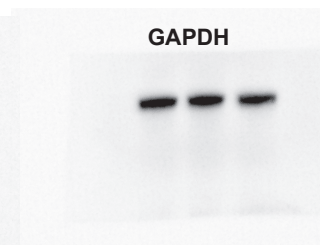

HepG2

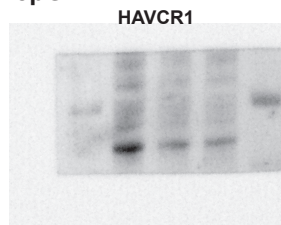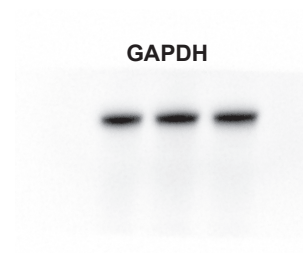

Supplement: Supplementary file 8 [file DataSheet_2.zip › raw data in vitro assay for edior checking/WB.pdf]
